# Selective Targeting of Class I Histone Deacetylases in a Model of Human Osteosarcoma

**DOI:** 10.3390/cancers13164199

**Published:** 2021-08-20

**Authors:** Haydee M. Torres, Ashley M. VanCleave, Mykayla Vollmer, Dakota L. Callahan, Austyn Smithback, Josephine M. Conn, Tania Rodezno-Antunes, Zili Gao, Yuxia Cao, Yohannes Afeworki, Jianning Tao

**Affiliations:** 1 Cancer Biology & Immunotherapies Group at Sanford Research, Sioux Falls, SD 57104, USA; Haydee.Torres@SanfordHealth.org (H.M.T.); amv5802@psu.edu (A.M.V.); rodeznoa@ualberta.ca (T.R.-A.); Yuxia.Cao@SanfordHealth.org (Y.C.); 2 Department of Chemistry and Biochemistry, South Dakota State University, Brookings, SD 57007, USA; 3 Medical Student Research Program, University of South Dakota, Vermillion, SD 57069, USA; Mykayla.L.Vollmer@coyotes.usd.edu; 4 Sanford Program for Undergraduate Research, University of Sioux Falls, Sioux Falls, SD 57104, USA; Dakota.Callahan2@SanfordHealth.org; 5 Sanford PROMISE Scholar Program, Harrisburg High School, Sioux Falls, SD 57104, USA; aesmithback@gmail.com; 6 Sanford Program for Undergraduate Research, Carleton College, Northfield, MN 55057, USA; josiemausconn@gmail.com; 7 Flow Cytometry Core at Sanford Research, Sioux Falls, SD 57104, USA; gaozili1989@hotmail.com; 8 Functional Genomics & Bioinformatics Core Facility at Sanford Research, Sioux Falls, SD 57104, USA; Yohannes.Tecleab@SanfordHealth.org; 9 Department of Pediatrics, Sanford School of Medicine, University of South Dakota, Vermillion, SD 57069, USA

**Keywords:** osteosarcoma, epigenetics, 4SC-202, domatinostat, histone deacetylase inhibitor, xenografts, osteoblast-like differentiation, transcriptome, signaling pathways

## Abstract

**Simple Summary:**

Osteosarcoma is the predominant form of primary bone cancer and outcomes for patients with metastatic disease have not improved for several decades. Recent genomic and pharmacological studies have implicated dysregulated histone deacetylases as druggable targets to treat several types of cancers, including osteosarcoma. This study aimed to assess the inhibitory effects of 4SC-202, a next-generation inhibitor for class I histone deacetylases, on human osteosarcoma cell growth in vitro and in vivo. We demonstrated that the anti-tumor effect of 4SC-202 involves combined cell-cycle arrest and apoptosis, as well as a reduction in cell invasion and migration. Moreover, 4SC-202 revised the global transcriptome and induced distinct signatures of gene expression in vitro. Additionally, 4SC-202 decreased tumor growth of established human tumor xenografts in immunodeficient mice in vivo. Our study suggests that 4SC-202 may be exploited as a valuable drug to promote more effective treatment of patients.

**Abstract:**

Dysregulation of histone deacetylases (HDACs) is associated with the pathogenesis of human osteosarcoma, which may present an epigenetic vulnerability as well as a therapeutic target. Domatinostat (4SC-202) is a next-generation class I HDAC inhibitor that is currently being used in clinical research for certain cancers, but its impact on human osteosarcoma has yet to be explored. In this study, we report that 4SC-202 inhibits osteosarcoma cell growth in vitro and in vivo. By analyzing cell function in vitro, we show that the anti-tumor effect of 4SC-202 involves the combined induction of cell-cycle arrest at the G2/M phase and apoptotic program, as well as a reduction in cell invasion and migration capabilities. We also found that 4SC-202 has little capacity to promote osteogenic differentiation. Remarkably, 4SC-202 revised the global transcriptome and induced distinct signatures of gene expression in vitro. Moreover, 4SC-202 decreased tumor growth of established human tumor xenografts in immunodeficient mice in vivo. We further reveal key targets regulated by 4SC-202 that contribute to tumor cell growth and survival, and canonical signaling pathways associated with progression and metastasis of osteosarcoma. Our study suggests that 4SC-202 may be exploited as a valuable drug to promote more effective treatment of patients with osteosarcoma and provide molecular insights into the mechanism of action of class I HDAC inhibitors.

## 1. Introduction

Osteosarcoma (OS) is the most common primary bone cancer that mainly occurs in children, adolescents, and young adults. In the past 40 years, the five-year survival rate has stagnated as standard patient care procedures, including multi-agent chemotherapy and surgery, have remained relatively unchanged [1]. In addition to the devastating side effects and social–emotional consequences of the rigorous treatment, additional complications include the need for prostheses after surgical amputation [2]. OS metastasis and chemoresistance are the key clinical factors leading to a low five-year survival rate of approximately 30% in patients with remote involvement [3,4,5,6]. Recent studies have shown that tumor suppressor and/or oncogene mutations, copy number alterations, fusion genes, and epigenetic dysregulation may lead to tumor formation, cancer metastasis, and multidrug resistance [7,8,9,10,11]. These distinct characteristics may pose as vulnerabilities that can be exploited as treatment targets. These findings require urgent investigations to explore the vulnerabilities outlined above in order to develop or repurpose second-line drugs that can improve patient survival.

Epigenetic dysregulation is an evolving hallmark of cancer and results from aberrant epigenetic regulators (such as writers, readers, and erasers) that modify histone and nonhistone proteins to balance the transcription of tumor suppressor genes and oncogenes [12,13]. Recently, aberrant, or dysregulated, epigenetic regulators are overwhelmingly discovered by next-generation sequencing studies in OS samples [5,6,14,15,16,17,18,19]. Targeting dysregulated erasers such as histone deacetylases (HDACs) can reinstate the epigenetic homeostasis from an abnormal epigenetic landscape and, as a result, they are emerging as druggable targets to treat several types of cancers, including osteosarcoma [1,6,11,20].

There are 18 unique human HDAC isoforms, which contain evolutionarily conserved catalytic domains that trace back to bacteria and yeast [21]. HDACs enzymatically remove acetyl groups (CH3CO-) from ε-amino groups of histone lysine residues within multi-protein complexes, which enables chromatin condensation and transcriptional repression and/or activation, and affects the stability and cellular location of non-histone proteins such as master transcription factor RUNX2, a key regulator of gene expression in normal bone and OS cells [22,23,24]. They can be categorized into four classes (I–IV) according to their domain organization, sequence, and functional similarity [25]. Class I retains four isoforms (HDACs 1, 2, 3, and 8), which are primarily located in the nucleus and are broadly expressed in most tissues. Class II retains six isoforms (HDACs 4, 5, 6, 7, 9 and 10), which shuttle between the cytoplasm and nucleus with a tissue-restricted pattern of gene expression. Class III retains seven isoforms (SIRTs 1–7), which may be located within the nucleus, cytoplasm, or mitochondria. Class IV retains one isoform (HDAC11), which is the least studied enzyme and shares similarities with the catalytic domains of classes I and II. Moreover, the catalytic function of classes I, II, and IV, dubbed the “classical” isoforms, depends on a cofactor zinc ion (Zn^2+^), whereas class III depends on the coenzyme nicotinamide adenine dinucleotide (NAD^+^) [22]. It is worth noting that small chemical inhibitors to HDACs have been designed and developed to chelate cofactor Zn^2+^ to prevent the accessibility of the active site from other complex components near the active site. These inhibitors can be classified into two groups. One is those with broad-spectrum inhibitory ability to HDACs, collectively named pan-HDAC inhibitors (or pan-HDACi), which can block the enzymatic function of two or more classes of isoforms. The other is those with an ability to selectively block one class or one isoform of HDACs, which are collectively named selective HDACi [26].

In skeletal development, HDACs promote endochondral and intramembranous ossification through regulating the expression of crucial genes and signaling pathways as well as mediating cell differentiation and survival of bone cells including osteoblasts, from which OS tumor arises [25,27]. For example, class I HDACs share 45–94% of amino acid sequence similarity, resulting in redundant cellular functions in development and adult tissue homeostasis. The co-expression pattern and overlapping roles of HDAC1 and HDAC2 have been reported in several cell types, including podocytes, cardiac, and osteoblasts [23,25,28,29,30]. In addition, HDAC1 and HDAC3 have been shown to physically interact with RUNX2 to regulate gene transcription in osteoblasts and chondrocytes [29,31]. The significantly elevated expression levels of HDAC1, HDAC2, and HDAC3 (HDAC1–3) have also been reported in several human OS cell lines and primary and metastatic tumor samples [11,32,33]. Therefore, the application of a selective inhibitor that can specifically target HDAC1-3 may lead to high-quality therapies with better efficacy and lower toxicity. 

Since FDA approval of vorinostat, the first HDACi, to treat patients with cutaneous T-cell lymphoma in 2006, several pan- and selective HDAC inhibitors have been developed and applied to the treatment of hematological malignancies [6,34]. Currently, HDACi drugs have not been approved for the treatment of solid tumors, including OS patients [6]. Additionally, a limited number of OS cases have been included in several completed and active clinical trials constructed for solid tumors, but no clinical trials have been specifically designed to study the disease (https://www.clinicaltrials.gov (accessed on 23 March 2021). For example, in a phase I trial study, only one OS patient was included and treated with a combination of vorinostat and bortezomib, but did not have a response [35]. A preclinical study using vorinostat showed modest inhibitory activity in OS cells and no objective responses for OS xenografts [36]. However, many more newly developed HDACi agents have recently been studied using established human OS cell lines. According to the chemical structures of HDACi, they can be categorized into four groups: (a) hydroxamic acids (e.g., vorinostat/SAHA [37,38,39,40,41,42,43,44,45], AR42 [46], trichostatin A/TSA [33,47,48,49,50,51,52,53,54,55], quisinostat [56,57,58], panobinostat/LBH589 [11,57,58,59,60], abexinostat [61], MC 1742 [62], and tubacin [63]), (b) short-chain fatty acids (e.g., valproate/VPA [64,65,66,67,68,69,70,71] and butyrate/NaB [72,73]), (c) benzamides (e.g., entinostat/MS-275 [74,75,76,77]), and (d) cyclic peptide (e.g., romidepsin/FK228 [11,58,60,78,79,80,81,82] and apicidin [80]). The latter two typically are considered selective HDACi. Hence, given the lack of clinical application and the paucity of information studying current HDACi in OS, the development and investigation of novel HDACi agents, especially selective-HDACi, are urgently needed.

4SC-202 (domatinostat), which is a recently developed small molecule that selectively targets class I HDACs, has been studied in several types of cancer, including urothelial carcinoma, squamous cell carcinoma, myelodysplastic syndrome, cutaneous T-cell lymphoma, cholangiocarcinoma, hepatocellular carcinoma, colorectal cancer, medulloblastoma, pancreatic cancer, and Merkel cell carcinoma [83,84,85,86,87,88,89,90,91,92,93]. Its safety and antitumor activity were established in a phase I study as a monotherapy for adult patients with hematological malignancies [94]. It is currently being tested in several clinical trials in the treatment of patients with hematologic malignancies and solid tumors (ClinicalTrials.gov Identifier: NCT04874831, NCT04393753, NCT04871594, NCT04133948 and NCT03812796). Prior to this study, 4SC-202 had not been examined for the treatment of OS. In our proof-of-concept study, we applied SJSA-1 and hFOB 1.19 cell lines to investigate global transcriptomic changes and drug effects of 4SC-202 in vitro and in vivo. We chose SJSA-1 because it is an established human OS cell line [95] that has been used to examine pan-HDACi drugs including, panobinostat and vorinostat [37,46,57,96], whereas hFOB 1.19 (hFOB) is an immortalized cell line derived from normal human fetal osteoblastic cells [97], which has been frequently applied in parallel to examine pan-HDACi including vorinostat, trichostatin A, and panobinostat [33,97,98].

## 2. Materials and Methods

### 2.1. Cell Culture and Treatment

For this study all cell lines, including human osteosarcoma SJSA-1 (CRL-2098) and the human immortalized osteoblast hFOB 1.19 (ATCC, CRL-11372), were purchased from the American Type Culture Collection (ATCC, Manassas, VA, USA). SJSA-1 and hFOB 1.19 were grown in a humidified chamber containing 5% CO_2_ and cultured in a growth medium (HyClone™ MEM-α medium, Marlborough, MA, USA, SH30265FS) containing 10% fetal bovine serum (Fisher Scientific, Waltham, MA, USA, ES009B) and 1% HyClone™ penicillin-streptomycin (Cytiva, SV30010). However, the hFOB 1.19 cell line media also contained 0.3 mg/mL G418 (Fisher Scientific, 10-131-035) and was maintained at 34 °C, as per ATCC protocol. Cells in 100 mm tissue culture dishes (Fisher Scientific, 430167) were treated with 1 μM 4SC-202 (Adooq Bioscience, Irvine, CA, USA, A14354-25) for 24 h unless differently stated. The corresponding amount of DMSO (vehicle) was used as a control. All assays were performed at 37 °C.

### 2.2. Western Blot Analysis

Western blotting analysis was performed as described previously [99,100]. Cells were briefly lysed with 1× Laemmli Sample Buffer solution (BioRad, Hercules, CA, USA, 1610737), boiled for 5 min to 95 °C, and sonicated. Lysates were separated on 4–20% Mini-PROTEAN^®^ TGX™ Precast Protein Gels (BioRad, 4561094) and transferred onto a PVDF membrane (BioRad, 1704272) using a semi-dry method of transfer (Bio-Rad Trans-blot Turbo system). The transferred blots were probed overnight with one of the following primary antibodies: rabbit anti-H3K4Me2 antibody (Diagenode, Denville, NJ, USA, C15200151), H3K27Ac (Diagenode, C15410196), rabbit anti-β-ACTIN (Li-Cor, 926-42210), JAG2 (Cell Signaling, #2205, C83A8), NOTCH3 (Proteintech, Chicago, IL, USA, 55114-1-AP), or NOTCH4 (Abcam, Cambridge, MA, USA, ab184742) primary antibodies. After incubation with IRDye 800 goat anti-rabbit (Li-Cor, Lincoln, NE, USA, 925-32211) and IRDye 680 goat anti-rabbit (Li-Cor, 925-68071) secondary antibodies (1:10,000) in 10% adult bovine serum blocking buffer was placed on a rocker for 1 h at room temperature. After several washes in 1XTBST, the protein signal was visualized on an Odyssey imaging system (Li-Cor, Lincoln, NE, USA) and quantified using β-actin as a normalizer with the optical density (OD) function of Image J software (NIH, Bethesda, MD, USA).

### 2.3. Cell Viability and Colony Formation Assays

The cell proliferation and viability assay was performed using a CCK-8 kit (Dojindo Molecular Technologies, CK04-11, Kumamoto, Japan) according to the manufacturer’s instructions and assays were performed at least three times. Cells were seeded into a 96-well plate (Fisher Scientific, N8010560) at a density of 6250 cells per well as five replicates. A black control was also used, which only contained media and no cells. Cells were treated with increasing concentrations of 4SC-202 (or vehicle) for 24, 48, and 72 h, with media and/or treatment replacement after 24 and 48 h for the latter time points, respectively. hFOB 1.19 was treated with increasing concentrations of 4SC-202 (or vehicle) for 72 h with media changes every 24 h. Once the wells reached the treatment time point, 10 μL of the CCK-8 was added to each condition, including negative controls, and incubated for 1 h at 37 °C in 5% CO_2_. The OD at 450 nm of each well was then read using a Cytation3. Statistical significance comparing each treated group to the control was analyzed using GraphPad software with one-way ANOVA (Holm method). For the colony formation assay [99,101], 1000 cells/mL were seeded into 6-well plates. After attachment to the wells, the cells underwent media changes with or without treatment with indicated concentrations of 4SC-202 every 2–3 days for about two weeks or until visible clonal colonies formed. The wells were washed with PBS, fixed with 10% formalin, and stained with 0.5% Crystal Violet solution at the endpoint of this study.

### 2.4. Wound Healing Assay 

To investigate 4SC-202-induced inhibition of cell migration, a wound-healing (i.e., in vitro scratch) assay was performed as we previously reported [99,100]. Cells were seeded into 6-well plates (Fisher Scientific, 353046) and grown to confluence. A vertical and horizontal cross-shaped scratch was made using a 2–2000 μL pipette tip on the monolayer of confluent cells. Dislodged cells and debris were removed by washing the cells three times with PBS. Fresh medium containing 1 µM 4SC-202 or DMSO vehicle was added (*t* = 0), and images were taken where the two scratch lines met using an upright Olympus IX71 microscope. After the indicated incubation time with either vehicle or drug treatment, the same area was photographed again. Scratched areas at the initial and final time points were quantified using the NIH ImageJ software (Bethesda, MD, USA). The percentage of relative wound healing was expressed according to the following formula: ((initial scratched area, 4SC-202 added) − (resulting scratched area, 4SC-202 added))/((initial scratched area, vehicle added) − (resulting scratched area, vehicle added)) × 100.

### 2.5. Boyden Chamber-Based Cell Migration and Invasion Assays

The in vitro Boyden migration and invasion assays were performed and modified using 8 μm pore-sized cell-culture inserts (Falcon, 08-771-21) into wells of a 24-well plate as previously reported [100]. Briefly, the cells were serum-started overnight with culture media containing either 4SC-202 or vehicle. Wells of a 24 well-plate were filled with culture medium containing 10% FBS prior to loading the cells at a concentration of 4 × 10^4^ cells/well into the upper compartment of the chamber (i.e., inside the cell-culture insert). For the invasion assay, the membrane at the bottom of the insert was coated with a layer of 0.2 mg/mL Matrigel overnight prior to the addition of the cells (Fisher Scientific, CB354248). After 24 h, the upper compartment was washed and fixed in 10% formalin. Cells that traveled through the insert remained and were visualized by staining with Crystal Violet (Fisher Scientific, C581-25). Any remaining cells on the upper surface of the insert were removed with a Q-tip, and the plate with inserts was then imaged. The dye from the cells was then extracted using 33% acetic acid (Fisher Scientific, A38S-212) and the OD was quantified on a Cytation3 at a wavelength of 570 nm. The OD of the extracted stain was used to determine the relative number of cells that invaded the gel barrier and passed through the insert’s pores.

### 2.6. Osteoblast Differentiation Assay

The osteoblast differentiation approach and quantification were completed according to the modified procedure described previously [100,102]. Cells were cultured to approach confluence in a 12-well plate (Fisher Scientific, 353043) and then treated with appropriate media. The osteoblast differentiation (OB diff) medium used contained 50 μM ascorbic acid (Cayman Chemical, Ann Arbor, MI, USA, 16457), 100 nM dexamethasone (Cayman Chemical, 11015), and 10 mM β-glycerophosphate (BGP) (Cayman Chemical, 14405). The assay was performed over 14 days, with a medium change every three days. At the endpoint, the cells were gently washed, fixed in 10% formalin, and stained to visualize calcium deposits with 40 mM Alizarin Red S (ARS) pH 4.2 (Sigma, St. Louis, MO, USA, A5533-25G). Images were taken with an upright microscope (Olympus, Center Valley, PA, USA, IX71). To quantify the staining, ARS was dissolved with 10% (*w*/*v*) cetylpyridinium chloride in 10 mM sodium phosphate (pH 7.0) on a rocker for approximately 3 h. Equal volumes of the extracted solution were measured on a Cytation 3 (BioTek, Crawfordsville, IN, USA) at 562 nm. Statistical significance comparing among groups was analyzed using GraphPad software with two-way ANOVA (Tukey method).

### 2.7. Analysis of Cell Cycle and Apoptosis by Flow Cytometry 

The cells were seeded into a 10 cm dish, allowed to reach 70% confluence, and then treated with DMSO vehicle or 1 µM 4SC-202 for 24 h. The media and adherent cells were pooled together into a single-cell suspension. For cell-cycle analysis, single-cell suspensions were pelleted and washed at room temperature twice with 1× phosphate buffered saline (PBS), fixed in 66% ethanol for 1 h, then rehydrated in 1× PBS. In order to ensure only DNA was being measured, the cells were incubated for 30 min with RNAse A (Qiagen, Germantown, MD, USA, 19101), followed by 1× PBS and 50 µg/mL propidium iodide in the dark. The PI was excited at 561 nm, the emission spectrum was detected through a 595 LP (longpass) mirror and 610/20 bandpass filter, and results were generated using a BD Fortessa system (Becton Dickinson, Franklin Lakes, NJ, USA). The data were analyzed using FlowJo v10.6 software (FlowJo, Ashland, OR, USA). For the apoptosis assay, single-cell suspensions were pelleted and washed twice with ice-cold 1× PBS and resuspended in 500 μL cold 1× binding buffer (25 mM HEPES, 1 mM EDTA, 2% FBS, 1% Pen/strep). After the cells were incubated with 5 μL Annexin V-FITC (Fisher Scientific, BDB560931) and 50 μg/mL propidium iodide (PI) for 15 min in the dark, they were analyzed by flow cytometry on a BD Fortessa system (Becton Dickinson, Franklin Lakes, NJ, USA). The FITC was excited at 488 nm and the emission spectrum was detected through a 495 LP (longpass) mirror and 530/30 bandpass filter, then analyzed by the BD FACSDiva v6.0 software. DMSO- and 4SC-202-treated groups were performed in triplicate with the following control groups: PI only, Annexin V-FITC only, and treatment only.

### 2.8. RNA Sequencing, Pathway Analysis, and Data Availability

Total RNA was isolated using a PureLink^®^ RNA Mini Kit (ThermoFisher Scientific, Waltham, MA, USA, 12183018A) according to the manufacturer’s instructions. RNA concentration and purity were measured by Thermo Scientific™ NanoDrop™ spectrophotometers. RNA integrity was measured on a Bioanalyzer 2100 with RNA 6000 Nano Labchips (Agilent Technologies Ireland, Dublin, Ireland). Twelve RNA samples with an RNA integrity number >8.0 were used for RNA cleanup, library preparation, and sequencing by Novogene according to the procedure and protocols of the company (Novogene Corporation, Sacramento, CA, USA). In short, according to the manufacturer’s protocol, 1 μg RNA was used for cDNA library construction using a NEBNext^®^ Ul-tra™ II RNA Library Prep Kit for Illumina^®^ (NEB #E7770). The mRNA was enriched with oligo(dT) beads, followed by two rounds of purification and random fragmentation by adding fragmentation buffer. We used random hexamer primers to synthesize the first-strand cDNA, and then added customized second-strand synthesis buffer (Illumina), dNTP, RNase H, and DNA polymerase I to generate the second strand (ds cDNA). After a series of end repair, polyadenylation, and sequencing linker connection, the double-stranded cDNA library was completed by size selection and PCR enrichment. The 250–350 bp insert library was quantified using a Qubit 2.0 fluorometer (Thermo Fisher Scientific, Waltham, MA, USA) and quantitative PCR. We used NGS3K to analyze the size distribution. Qualified libraries were sequenced on an Illumina platform using a paired-end 150 run (2 × 150 bases). The high-quality reads from 27.5 to 31.5 million were generated from each library. Paired-end reads were aligned to the human genome (GRCh37) and annotated with GENCODE gene annotation (v32) using STAR.

Raw counts were estimated with the option set (--quantMode GeneCounts) in STAR. Differential expression (DE) analysis were performed using the R [103] package DESeq2 [104]. DE genes were identified based on cutoff values of 0.05 for adjusted *p*-value and/or log2-fold-change. To account for gene length bias, gene ontology and KEGG pathway enrichment analysis were conducted using R package goseq [105]. The Benjamini–Hochberg correction for multiple testing in enrichment was used and pathways with adjusted *p*-values less than 0.05 were declared significant [106]. The principal component analysis (PCA) plots and heatmaps of Euclidean distances among samples are based on the expression data and using DESeq2 [104]. Heatmaps of the top varying genes in each experiment were produced using the R package pheatmap [107]. The number of fragments per kilo base per million mapped reads (FPKM) was calculated for each sample to help visualize expression patterns for each unigene between the treated and untreated samples. Additional functional annotation, pathways, and gene network analyses were performed by IPA (Ingenuity Pathways Analysis, http://www.ingenuity.com/ (accessed on 11 December 2020) with default parameters. 

All RNA-seq experiments were performed in at least three biological replicates under each condition. The raw sequence data of RNA-seq generated in this study are stored in the National Center for Biotechnology Information (NCBI) sequence reading archive database (accession numbers: SRR14772115 to SRR14772126). All other data can be obtained from the corresponding author upon reasonable request.

### 2.9. In Vivo Studies 

Athymic nude female mice (stock #002019) were purchased from Jackson Laboratory (Bar Harbor, ME, USA). Before transplant, SJSA-1 cells were harvested, washed, and resuspended in serum-free MEM-alpha media. Nude mice (6–8 weeks old) were anesthetized by isoflurane inhalation (3%). Mice were injected subcutaneously to the rear flank with 250 μL of cell suspension (5 × 10^6^ cells/injection) using a 27G needle (*n* = 16). Once the tumor size reached an average volume of around 60 cubic millimeters, the mice were randomly divided into two groups, control and treatment, and treated with either vehicle (10% DMSO + 45% PEG400) or 4SC-202 (50 mg/kg/d) via intraperitoneal injections daily for 16 days. Mouse body weight was measured daily. Tumor size was measured using digital calipers, and volumes were calculated according to the formula, tumor volume (cubic millimeter) = (Width × Width × Length)/2. The mice were housed in a specific pathogen-free facility under controlled conditions of light, temperature, and humidity.

### 2.10. Statistical Analysis 

Data were analyzed using Student’s *t*-test, or one-way (Holm method) or two-way ANOVA (Tukey method) accordingly (GraphPad Prism, GraphPad Software, Inc., La Jolla, CA, USA). A *p*-value less than or equal to 0.05 was considered statistically significant. All data unless otherwise specified are expressed as mean ± standard deviation.

## 3. Results

### 3.1. HDAC1-3 Have the Highest Expression among All Isoforms in Human OS Cells

**Figure 1:**
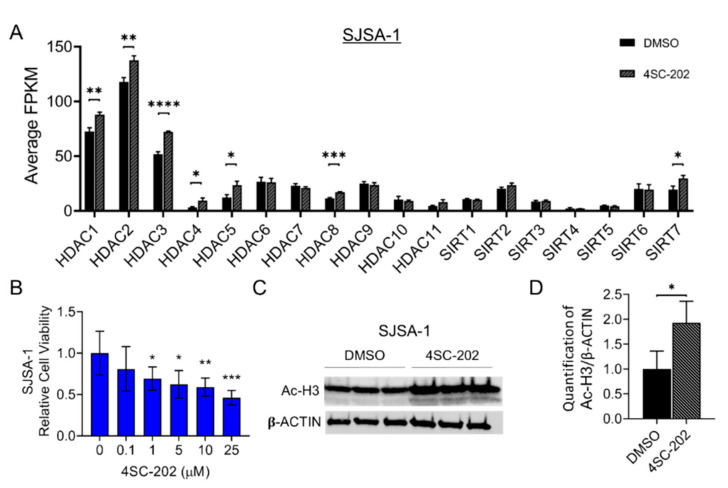
Impact of selective pharmacological inhibition of class I histone deacetylases with 4SC-202 on human SJSA-1 OS cells. (**A**) Gene expression of 18 histone deacetylases (HDACs) in SJSA-1 cells. Average FPKM (i.e., fragments per kilo base per million mapped reads) values generated from RNA-seq analysis of indicated genes from 3 samples treated with DMSO vehicle (dark bar) and 3 samples treated with 4SC-202 (grey bar). (**B**) 4SC-202 effect on cell proliferation and viability treated with increased concentration of 4SC-202 for 24 h. Statistical significance comparing each treated group to the control was analyzed using GraphPad software with one-way ANOVA (Holm method). (**C**) Representative Western blot analysis of Ac-H3 and β-ACTIN in DMSO-treated and 4SC-202-treated conditions in OS cells. (**D**) Densitometric quantification of the blots of protein band intensity of Ac-H3 normalized to housekeeping β-ACTIN bands. Number of asterisks indicates level of statistical significance between groups. * *p* < 0.05, ** *p* < 0.01, *** *p* < 0.001, **** *p* < 0.0001. Data are presented as mean (SD).

**Figure 2:**
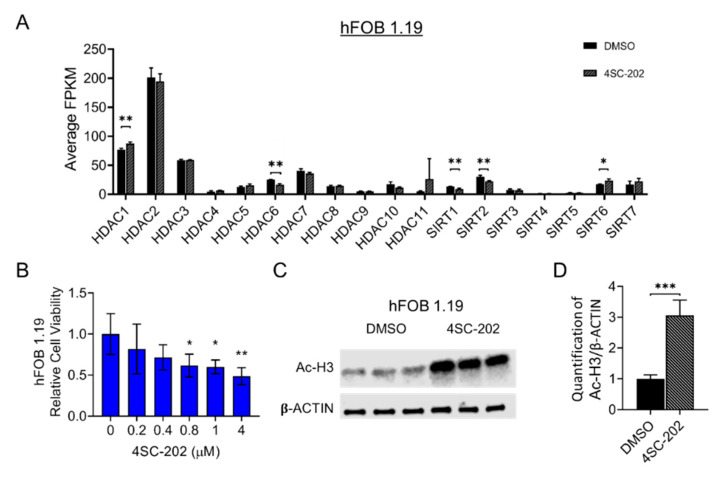
Impact of selective pharmacological inhibition of class I histone deacetylases with 4SC-202 on human hFOB 1.19 cells. (**A**) Gene expression of 18 histone deacetylases (HDACs) in hFOB 1.19 cells. Average FPKM (i.e., fragments per kilo base per million mapped reads) values generated from RNA-seq analysis of indicated genes from 3 samples treated with DMSO vehicle (dark bar) and 3 samples treated with 4SC-202 (grey bar). (**B**) 4SC-202 effect on cell proliferation and viability. Statistical significance comparing each treated group to the control was analyzed using GraphPad software with one-way ANOVA (Holm method). (**C**) Representative Western blot analysis of Ac-H3 and β-ACTIN at DMSO-treated and 4SC-202-treated conditions in bone cells. (**D**) Densitometric quantification of the blots of protein band intensity of Ac-H3 normalized to housekeeping β-ACTIN bands. Number of asterisks indicates level of statistical significance between groups. * *p* < 0.05, ** *p* < 0.01, *** *p* < 0.001. Data are presented as mean (SD).

To have a clearer understanding of the relative expression levels of class I HDACs, other HDAC isoforms, and the status of gene expression in the entire transcriptome of untreated cells, we first performed a next-generation RNA sequencing (RNA-seq) analysis. We found that HDAC1, HDAC2, and HDAC3 have much higher gene expression than other isoforms in SJSA-1 OS cells, as well as hFOB cells (Figure 1A and Figure 2A). Previous research showed that 4SC-202 is a selective benzamide-type HDACi-targeting class I HDAC (Figure S1A,B) with a higher specificity (lower inhibitory constant (Ki) values) for HDAC1 (14.8 nM), HDAC2 (38.8 nM), and HDAC3 (27.9 nM) than other isoforms and lysine-specific histone demethylase 1A (LSD1) (Ki values > 1800 nM) [94]. These data prompted us to further examine the potential therapeutic effects of this newly developed drug in human OS cells. Thus, our data suggest that SJSA-1 together with hFOB are the prominent candidate cell lines to examine the repressive consequences of 4SC-202. 

### 3.2. 4SC-202 Impairs Human Osteosarcoma Cell Growth and Clonogenicity In Vitro

To investigate the anti-proliferative effects of 4SC-202 in OS in vitro, we cultured SJSA-1 cells in the presence of different concentrations (0.1 to 25 μM) of 4SC-202. Treatment with 4SC-202 resulted in a dose-dependent inhibitory effect on cell growth at 24 h (h) (Figure 1B). Among five concentrations, 1 μM or higher of the drug was enough to suppress the cell viability and proliferation in a time-dependent fashion (24, 48, and 72 h) (Figure S1C,D). Moreover, a similar effect was observed in hFOB 1.19 cells that could be attributed to their high proliferation rate (Figure 2B and Figure S1E,F). To assess the activity of 4SC-202 in altering acetylation and methylation of histone proteins, we cultured the cells in the absence or presence of 1 µM 4SC-202 for 24 h and then harvested them for Western blotting analysis to detect acetylated histone 3 (Ac-H3), a substrate of HDACs, and demethylated H3K4Me2, a substrate of LSD1. As shown in Figure 1 and Figure 2, Ac-H3 protein expression was significantly increased, whereas a change in H3K4Me2 expression was not detected in SJSA-1 cells (Figure 1C,D and Figure S2E,G) or hFOB cells (Figure 2C,D and Figure S2F,H). We also observed an increase in H3K27Ac protein level in a dose-dependent manner to respond to 4SC-202 treatment in SJSA-1 and hFOB cells (Figure S2A–D). Notably, the mRNA levels of class I HDACs in the presence of 1 µM 4SC-202 for 24 h were also altered, likely due to a compensatory mechanism (Figure 1A and Figure 2A). Our data suggest that 4SC-202 impairs human osteosarcoma cell growth in vitro through the inhibition of the activity of HDACs.

**Figure 3:**
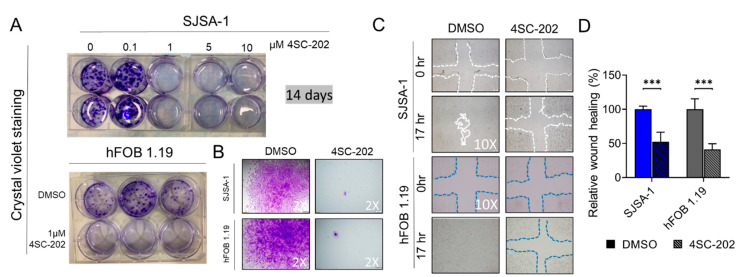
Effect of 4SC-202 on colony formation and wound healing. (**A**) Representative images of crystal violet-stained colonies in wells from SJSA-1 (**top** panel) and hFOB 1.19 cells (**bottom left**). Cells were treated with indicated concentrations of 4SC-202 or DMSO vehicle for 14 days. (**B**) Representative images of colonies under microscope with 2× objective magnification. (**C**) Representative images of wound healing at indicated hours after the mechanical scratch under 10× objective magnification from SJSA-1 (**top** panel) and hFOB 1.19 cells (**bottom**). **Left**: vehicle, **right**: treated with 4SC-202. The white and blue lines indicate the edges of the wounded area. (**D**) Quantitative analysis of the wound healing area after the scratch. Wound healing area of vehicle-treated cells is defined as 100% versus areas of drug-treated cells (*** *p* < 0.001). Data are presented as mean (SD).

To examine the long-term effect of 4SC-202 on cell growth and clonogenicity in vitro, we performed a colony formation assay, which can detect the ability of a single cell to grow into a colony (>50 cells) by clonal expansion under drug selection. Crystal violet staining clearly showed that a 14-day treatment with 4SC-202 at concentrations ranging from 1 to 25 μM significantly inhibited proliferative SJSA-1 colonies, indicating an inhibitory effect on cell growth and clonogenicity (Figure 3A). Notably, we also observed remnant cells or small clones (<50 cells), which implies cancer cell survival and drug resistance after treatment with 4SC-202 at this concentration (Figure 3B). Moreover, the clonogenicity of hFOB 1.19 cells was also strongly inhibited by 1 μM 4SC-202 treatment for 14 days (Figure 3A,B). Since clonogenic activity is a sensitive indicator of capacity for renewal of cancer stem cells (CSCs) and drug resistance, this result implies that HDACs may be required for maintenance of CSC numbers and resistance of chemotherapy drugs in OS tumor tissues. Altogether, 1 μM 4SC-202 was an effective concentration in both cell lines and was therefore applied for all further experiments in this study.

### 3.3. Cell Migration and Invasion In Vitro Are Partially Suppressed by 4SC-202

**Figure 4:**
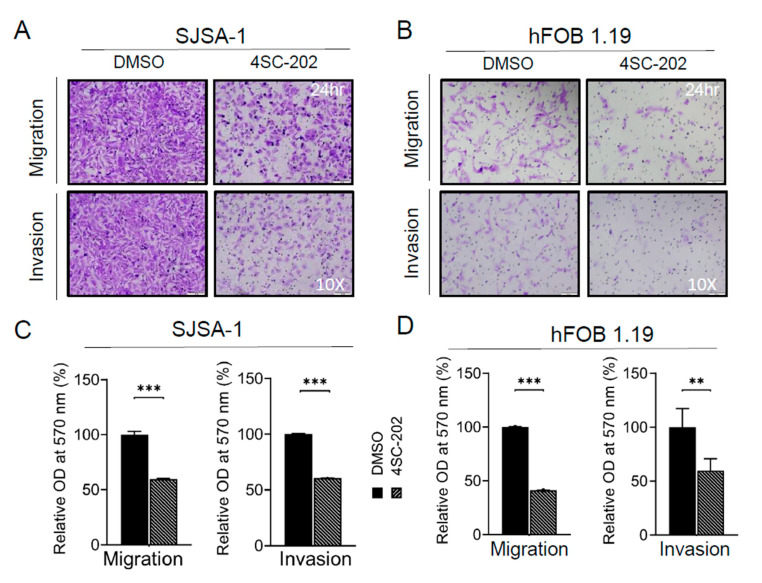
Effect of 4SC-202 on cell migration and invasion. (**A**) Representative images of crystal violet-stained SJSA-1 cell migration (top panel) or invasion (bottom panel) in a trans-well system with or without 4SC-202 treatment under 10× objective magnification. (**B**) Representative images of crystal violet-stained hFOB 1.19 cell migration (top panel) or invasion (bottom panel) in a trans-well system treated with or without 4SC-202 under 10x objective magnification. (**C**) Quantification of panel A data. (**D**) Quantification of panel B data. ** *p* < 0.01, *** *p* < 0.001. Data are presented as mean (SD).

To dissect the effects of 4SC-202 on cell migration, we first performed a wound-healing assay. After 17 h, 4SC-202-treated SJSA-1 and hFOB 1.19 cells migrated significantly less than those treated with vehicle DMSO control (Figure 3C,D). A Boyden chamber-based cell migration system was applied to further examine this phenomenon of migration suppression under 4SC-202 treatment. Cells were serum-starved for 16 h to minify the contribution from cell proliferation before seeding into the trans-well chamber. Quantification of cells able to travel through the pores showed an approximate 55% decrease in 4SC-202-treated cells compared to vehicle-treated cells (Figure 4A–D). A layer of Matrigel was added to the top insert of the chamber to test the cell ability to invade through the gel under 4SC-202 treatment. We found that 4SC-202 significantly decreased the invasive ability of both SJSA-1 and hFOB 1.19 cells by approximately 40% compared to that of the cells treated with vehicle (Figure 4A–D). Altogether, this data implies that inhibition of class I HDACs by 4SC-202 may suppress tumor metastasis in human OS patients. 

### 3.4. Cell Cycle Is Arrested at the G2/M-Phase Checkpoint by 4SC-202

**Figure 5:**
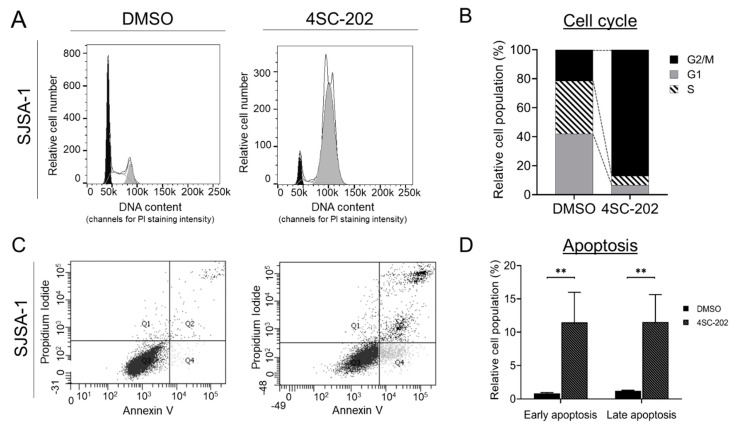
Effect of 4SC-202 on the cell-cycle distribution and apoptosis in human SJSA-1 OS cells. (**A**) Representative cell-cycle flow cytometry profiles of cells treated with or without 4SC-202 for 24 h. The DNA content of cells was analyzed by flow cytometry after staining with propidium iodide. (**B**) Relative cell population quantified data from cell-cycle profiles in A. (**C**) Representative flow cytometry scatter plots of cells treated with or without 4SC-202 for 24 h and stained with annexin V (positive for apoptotic cells) and propidium iodide. (**D**) Quantification of apoptotic profiles for relative cell population from C. ** *p* < 0.01. Data are presented as mean (SD).

HDACi agents are known inducers of cell-cycle arrest and apoptosis in many cancer cell types [108]. A dose-dependent disturbance of cell-cycle progression by 4SC-202 was observed in several cancer types [83,84,85,93]. To examine the cell-cycle behavior in human OS cells, unsynchronized SJSA-1 cells were incubated in the absence or presence of 1 μM 4SC-202 for 24 h before propidium iodide (PI) staining. Our flow cytometry analysis demonstrated that the percentage of cells at the G2/M phase in drug-treated cells significantly increased to 87.08% from 21.49% compared to vehicle control, whereas cells at the G1 and S phases in 4SC-202-treated cells decreased to 6.66% and 6.26%, respectively, and from 41.96% and 36.55%, respectively, in control (Figure 5A,B). In parallel, we also observed a dramatic blockage of the cell cycle in hFOB cells (Figure S3A,B). To further explore the anti-tumor growth effects of 4SC-202 in OS cells, we performed flow-cytometric quantification of an apoptotic response using annexin V and PI double staining on cells treated with the drug for 24 h. Treatment with 4SC-202 significantly increased the population of early-phase (Q4 quadrant: annexin V^+^ PI^−^) and late-phase (Q2 quadrant: annexin V^+^ PI^+^) apoptotic cells to 10.63% and 10.33%, respectively (Figure 5C,D). Notably, we observed no significant cell death (Q1 quadrant: Annexin V^-^ PI^+^) in treated SJSA-1 cells compared to the control. Moreover, 4SC-202 treatment showed a similar drug effect on hFOB cells, although a slightly different pattern with an increased population of early- and late-phase apoptotic and dead cells to 4.9%, 19.16%, and 9.13%, respectively (Figure S3C,D). Collectively, these data indicate that cell-cycle arrest and apoptosis induced by 4SC-202 may be responsible for the acute anti-proliferative effect of the drug. 

### 3.5. 4SC-202 Is Incompetent at Initiating and Enhancing Osteogenic Differentiation of Osteosarcoma Cells into Mineralizing Osteoblast-Like Cells

**Figure 6:**
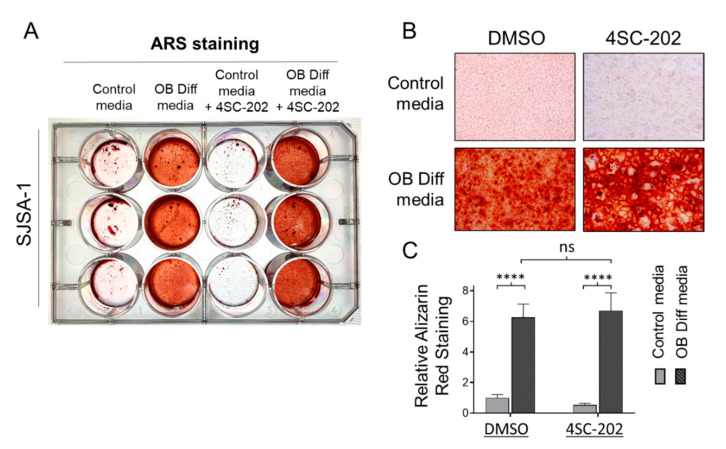
Effect of 4SC-202 on osteoblast-like differentiation and mineralization. (**A**) Representative photo of 12-well plate wells stained with Alizarin Red S after treatment for two weeks with control media or osteoblast differentiation (OB Diff) media or control media + 4SC-202 or OB Diff media + 4SC-202. (**B**) Representative images of the plate wells in A under 10× objective magnification. (**C**) Quantitative results of Alizarin Red S staining in all 4 groups in A. **** *p* < 0.0001 (two-way ANOVA with Tukey method). Data are presented as mean (SD).

Since osteosarcoma cells habitually possess an osteogenic differentiation program, we probed the possibility of 4SC-202 alone in promoting osteoblast-like differentiation (OB diff) phenotype. As shown in the left panels of Figure 6A–C and consistent with our previous study [100], SJSA-1 cells cultured with OB diff medium had a strong ability to differentiate into mature mineralizing cells to produce bone matrix and calcium (positively stained by Alizarin Red S). In the absence of OB diff agents, we found that 1 μM 4SC-202 treatment was incompetent at inducing osteogenic differentiation for 14 days (Figure 6A). Furthermore, with the addition of 1µM of 4SC-202 to the OB diff medium, we found that SJSA-1 cells produced similar mineralized nodules (Figure 6B) and content of calcium to cells treated with OB-diff alone (Figure 6C). These data indicate that 4SC-202 has little capacity to promote osteogenic differentiation.

### 3.6. In Vitro 4SC-202 Treatment Revises Global Transcriptomic Profiling and Induces Distinct Gene Expression Signatures in Human Osteosarcoma Cells

**Figure 7:**
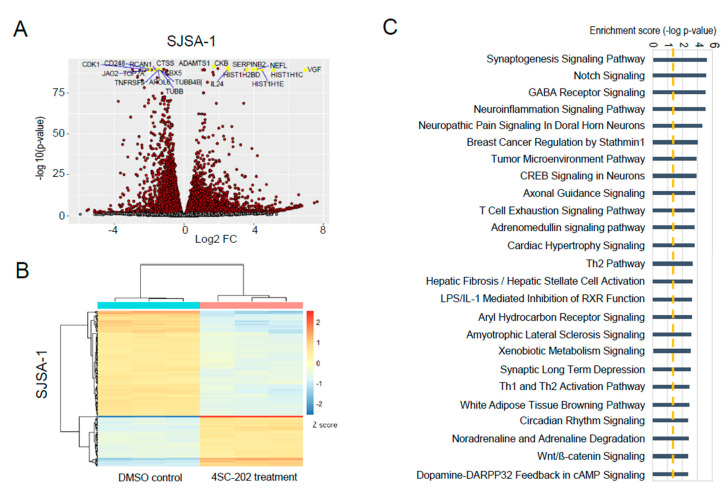
Differentially expressed genes and pathways in human OS cells. (**A**) A volcano plot of gene expression from RNA-seq analysis between the vehicle control and 4SC-202-treated SJSA-1 OS cells. The top 20 significantly differentially expressed genes are labeled. (**B**) Unsupervised hierarchical cluster analysis and heatmap representation of differentially expressed genes in SJSA-1 cells treated with either 1 µM 4SC-202 or DMSO for 24 h. Intensity of color indicates expression levels (red, high; blue, low). Each column indicates a distinct sample, and each row indicates an individual gene. (**C**) Ingenuity pathways analysis (IPA) of cancer canonical signaling pathways associated with significantly regulated genes (*p* < 0.05) in 4SC-202-treated samples compared to the vehicle-treated samples.

To assess 4SC-202-induced alterations in global mRNA expression and gene signature, we performed whole transcriptomic profiling using an unbiased next-generation sequencing approach for SJSA-1 and hFOB cells. For this purpose, total RNA was extracted from cells treated for 24 h with 1 µM 4SC-202 or 0.1% DMSO and then subjected to shotgun RNA sequencing (RNA-seq) analysis. All samples showed an RNA Integrity Number (RIN) higher than 9.1 on a bioanalyzer, confirming the RNA integrity and quality (Figure S4A,B). Principle component analysis of RNA-seq data revealed a marked separation of gene expression changes between the control and 4SC-202 treatment groups for each cell line (PC1, 97% for both) and a low level of variability among biological replicates (in triplicate) (PC2, 1% and 2% for SJSA-1 and hFOB, respectively) (Figure S5A,B). Heatmaps of Euclidean distances between samples further substantiated the similarity of gene expression between samples from the same group (i.e., treated or untreated) as well as the differences between samples from different groups (i.e., treated versus untreated) (Figure S6A–C). 4SC-202 treatment profoundly changed the global transcriptome of the SJSA-1 and hFOB cells, as indicated by the total number of significant differentially expressed genes (DEGs) given in Tables S1 and S2. Approximately half of DEGs were common to the two cell lines (Figure S5E). Among them, the top 20 differentially expressed genes with the smallest *p*-value are labeled with gene symbols (Figure 7A and Figure S6A). The top 200 significantly upregulated or downregulated genes in the drug-treated groups are highlighted in Figure 7B and Figure S6B. Ingenuity pathway analysis (IPA) of the differentially expressed genes in the 4SC-202-treated compared to control cells highlighted that the human OS cells and transformed osteoblasts shared altered canonical signaling pathways, including Wnt/beta-catenin, cAMP-mediated signaling, calcium signaling, eNOS signaling, VDR/RXR activation, bladder cancer signaling, tumor microenvironment pathway, breast cancer regulation by Stathmin1, synaptogenesis signaling, pathway regulation of the Epithelial-Mesenchymal Transition (EMT) pathway, and the role of osteoblasts, osteoclasts, and chondrocytes in rheumatoid arthritis (Figure 7C, Figure S5F and Figure S6C, Tables S3 and S4).

**Figure 8:**
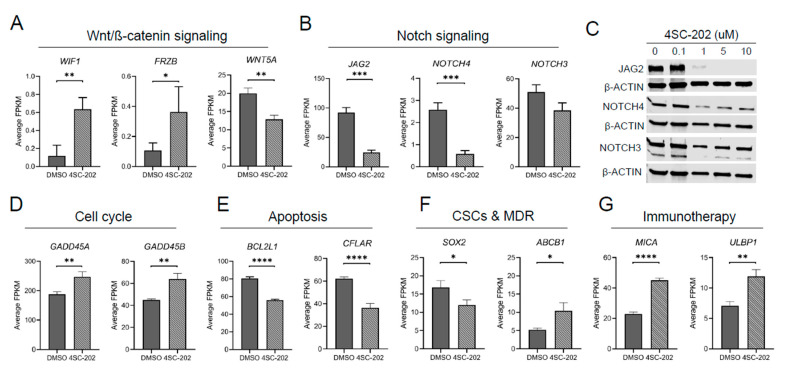
Candidate genes regulated by 4SC-202 in human SJSA-1 OS cells. Selected differentially expressed genes in (**A**) the Wnt/ß-catenin signaling pathway and (**B**) the Notch signaling pathway. (**C**) Representative Western blot analysis of JAG2, NOTCH4, NOTCH3, and β-ACTIN in OS cells treated with or without 4SC-202. Selected differentially expressed genes in (**D**) cell cycle, (**E**) apoptosis, (**F**) cancer stem cells (CSCs) and multidrug resistance (MDR), and (**G**) immunotherapy. Average FPKM values from RNA-seq analysis were used. * *p* < 0.05, ** *p* < 0.01, *** *p* < 0.001, **** *p* < 0.0001. Data are presented as mean (SD) of triplicate samples treated with or without 4SC-202.

On the other hand, we uncovered that the Notch signaling pathway was significantly altered only in SJSA-1 cells (Figure 7C). Because Notch and Wnt signaling are two of the most deregulated cancer signaling pathways in our data, we investigated individual genes that were significantly regulated upon 4SC-202 treatment. We found that Wnt antagonists *WIF1* and *FRZB* were upregulated but *WNT5A* was downregulated. We also found that JAG2 and NOTCH4 mRNA and protein levels were significantly decreased upon the drug treatment (Figure 8B,C). Although NOTCH3 mRNA levels were not significantly altered, we found a significant decrease in its protein levels upon increasing concentrations of 4SC-202 (Figure 8B,C). Interestingly, we observed several significantly dysregulated key genes involved in cellular processes and cancer treatment, including cell cycle (*GADD45A* and *GADD45B* in Figure 8D), apoptosis (*BCL2L1* and *CFLAR* in Figure 8E), stem-cell renewal and drug resistance (SOX2 and ABCB1 in Figure 8F), and immunotherapy (*MICA* and *ULBP1* in Figure 8G). This implies that 4SC-202 induces distinct gene expression signatures in treated SJSA-1 OS cells. 

### 3.7. In Vivo 4SC-202 Treatment Reduces the Tumor Growth of Osteosarcoma in Mice

**Figure 9:**
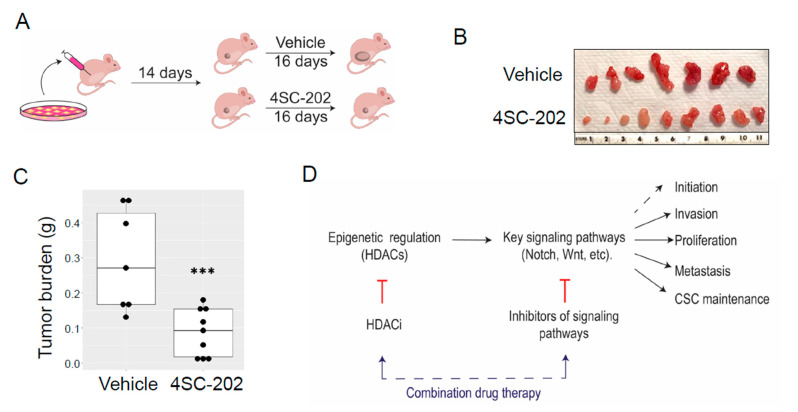
4SC-202 reduces the tumor growth of SJSA-1 cell in vivo. (**A**) Illustration of in vivo experimental procedure using a xenograft model of nude mice. (**B**) Photographs of SJSA-1 xenografted tumors at harvest from nude mice. (**C**) Quantification of tumor burden of xenografted tumors treated with or without 4SC-202 (*n* = 7 for vehicle and *n* = 9 for 4SC-202 treated mice, *** *p* < 0.001, data are presented as mean (SD)). (**D**) Proposed future studies on tumor cell behaviors of osteosarcoma.

To assess the therapeutic potential of 4SC-202 on established human tumor xenografts in immune-compromised mice, we implanted SJSA-1 cells into the flanks of athymic nude mice and allowed tumors to grow for about two weeks until detectable (Figure 9A). The mice were then randomly divided into two groups and treated daily with either vehicle or 4SC-202 for 16 days (Figure 9A). Consistent with the observed in vitro effects, gross observation of the harvested tumors indicated a lessened tumor size in the 4SC-202-treated group (Figure 9B). Compared to the vehicle mice, the average tumor mass of the treated group was significantly reduced by 70.56%. At the same time, no obvious body weight loss or pathological changes were observed during this treatment period with this dosage of 50 mg/kg, which was used in a previous study in treatment of bile duct cancer [86]. It is also worth noting that, consistent with another previous study using the SJSA-1 OS model in nude mice [109], no metastasis was observed in either group of mice.

## 4. Discussion

Similar to “oncogene addiction,” the hypothesis of “epigenetic vulnerability of cancer cells” is evolving into a new axiom, which has been endorsed by increasing evidence from studies on HDACs and HDACi [6,20]. Compared with normal cells that have multi-tiered and redundant compensating epigenetic pathways or factors, some cancer cells mainly rely on specific epigenetic pathways or factors such as class I HDACs to sustain the function of key genes to maintain cell survival, growth, invasion, metastasis, and drug resistance. This study on the use of a selective HDACi, 4SC-202, against HDAC1-3 provides new evidence to support this hypothesis in osteosarcoma.

Previous studies have shown that 4SC-202 has an anti-tumor effect against several types of hematologic malignancies and solid tumors [83,84,85,86,87,88,89,90,91,92,93]. Our study adds osteosarcoma to this catalog. Figure 1C and Figure 2C align with the previous findings that 4SC-202 effectively targets HDAC1-3 but not LSD1 [83,94,110]. Consistent with the significantly increased expression in primary human osteosarcoma tissues [32,33], this study reports higher expression of HDAC1-3 in human SJSA-1 OS cells (Figure 1A). A study using pan-HDACi (panobinostat) and a HDAC1/2 selective inhibitor (romidepsin) demonstrated that the combined functions of HDAC1 and HDAC2 may contribute to the maintenance of osteosarcoma growth and metastasis [11]. Other studies using a HDAC2 selective inhibitor (CAY10683) and a HDAC1/3 selective inhibitor (MS-275) further showed that individual HDAC2 or HDAC3 may also contribute to OS behaviors [74,77,111]. Moreover, HDAC1 may have additional unique roles in OS progression and drug resistance [47,53,72]. Our findings support the aforementioned studies on the role of HDAC1-3 in OS cell proliferation, invasion, metastasis, and cancer stem cell (CSC) maintenance (Figure 9D). If the essential roles of HDAC1-3 in OS cells hold true, it is assumed that 4SC-202 may supersede the aforementioned pan-HDACi, which have systemic toxicity [57], and some selective HDACi with a narrower selection, which may have limited clinical utility [81]. Indeed, a study showed that the cardiotoxicity by aselective class I HDACi may be less than that of pan-HDACi and other selective HDACi due to fewer alterations in the expression of heart-specific genes [112]. However, there are 11 HDACs from classes I, II, and IV. The substrate specificity of each HDAC and the requirements for pan-HDACi and/or selective HDACi are still contestable. The HDACi that can achieve the safest and most effective therapeutic effects still needs to be determined. However, some concerns remain in considering the clinical use of highly selective HDACi, including 4SC-202, in OS patients given the yet unclear roles of HDAC1-3 as putative tumor suppressors in human osteosarcoma formation and their well-defined roles in the maintenance of in vivo bone mass and homeostasis [32,113]. Nevertheless, results from this in vitro and in vivo study using 4SC-202 imply an oncogenic role of HDAC1-3 in OS cells and suggest future preclinical studies for its combination therapy with other molecular-targeted agents such as inhibitors of signaling pathways (Figure 9D). 

Across all HDAC inhibitors, induction of G1/S phase cell cycle arrest is dominant over a G2/M arrest [26]. We found that 4SC-202 induced G2/M cell cycle arrest and apoptosis in SJSA-1 OS cells, which is consistent with the latest studies in other cancer types using this agent [83,84,85,93]. A G2/M cell cycle arrest is reported to be highly dependent on the upregulation of GADD45s, whereas a G1 arrest is considered to be highly dependent on the upregulation of p53/CDKN1A [48]. Our finding that 4SC-202 significantly increased mRNA levels of GADD45A and GAD45B, whereas p53 expression was significantly decreased, provides a potential molecular mechanism of action of 4SC-202 (Figure 8D and data not shown). We further provide a molecular basis for treatment-induced apoptosis of OS cells by showing a decrease in well-known anti-apoptosis genes (BCL2L1 and CFLAR/c-FLP) (Figure 8E). This is consistent with previous studies that reported that expression of these two genes can be suppressed by various HDACi in cancer cells [76,78,82,114]. 

Notably, some studies using SJSA-1 OS cells and the non-cancerous cell line hFOB as benign control cells found that they are not sensitive to a pan-HDACi, SAHA [37,46,98]. In this study, we observed a strong inhibition of proliferation, colony formation, invasion, migration, cell cycle, and cell survival after treatment with 4SC-202 in both cell lines at comparable concentrations (Figure 1, Figure 2, Figure 3, Figure 4 and Figure 5 and Figures S1–S3). In keeping with our observations, this phenomenon was reported in 4SC-202-treated urothelial cancer cells and HEK-293 cells, a urothelial benign control cell line [85]. Indeed, more recent studies have shown that both SJSA-1 and hFOB cells are very sensitive to the pan-HDACi panobinostat (10–15 nM) [57,59]. It is not surprising that hFOB cells immortalized by SV40 TAg (large T antigen) behave like mesenchymal-stromal cells (MSCs) [115,116]. Furthermore, low-dose panobinostat has been shown to act predominantly as a potent “differentiating” agent that drives terminal osteoblast-like differentiation in OS cells [57]. Nevertheless, we showed that 4SC-202 cannot initiate and enhance osteoblastic differentiation of OS cells, indicating that 4SC-202 and panobinostat may have different effects and/or targets on osteoblast-like differentiation preprogram (Figure 6). Together, our data support the concept that 4SC-202 is a strong inhibitor of proliferating cells but may be not a “differentiation agent”.

Different mechanisms have been proposed for how 4SC-202 exerts its effects on tumor growth and survival. These include suppressing oncogenic hedgehog-GLI signaling in medulloblastoma cells [89,117], inhibiting NF-κB pathway signaling in myelodysplastic syndrome cells [84], activating the ASK1/Cyp-D mitochondrial pathway in hepatocellular carcinoma cells [92], promoting epithelial gene expression of BRD4 and MYC nuclear cofactors in pancreatic cells [88], disabling microtubules and thereby directly affecting mitotic spindle formation [83], and restoring immunogenic HLA class I surface gene expression on Merkel cell carcinoma cells [93]. Our findings suggest that 4SC-202 has the potential to impair tumor growth and metastasis by directly suppressing Wnt and Notch signaling pathways in OS cells (Figure 8C and Figure 9A–C). Previously, our study and others have suggested that dysregulated Wnt and Notch pathways contribute to osteosarcoma initiation, progression, and metastasis [5,8,99,109,118,119,120,121]. Notably, a recent study highlighted the essential roles for HDAC1–3 in chromatin regulatory complexes of rhabdomyosarcoma pediatric tumors, and that HDACi-induced hyperacetylation disrupts key interactions at super enhancers, resulting in decreased transcription at super enhancer core regulatory transcription factor genes. It is possible that this is a conserved HDACi mechanism, and thus that this is occurring with 4SC-202-induced hyperacetylation as indicated by protein levels of acetylated histone 3 (Figure 1C and Figure 2C, and Figure S2). Further studies are needed in order to unravel the core regulatory transcription factor circuitry in OS and how 4SC-202 may deregulate these phenomena.

Earlier studies using various OS cells found that treatment with a pan-HDACi or selective HDACi affects the gene expression of many components in Wnt and Notch pathways, such as β-catenin and Notch1 [41,42,59,60,122,123]. In this study, we uncovered that 4SC-202 significantly affects several key components in Wnt and Notch pathways by either restoring (e.g., WIF1 and FRZB) or suppressing their expression (e.g., WNT5A, JAG2, NOTCH4 and NOTCH3) in OS cells (Figure 8A,B). Interestingly, we previously showed that WNT5A is highly expressed in most human OS tissues [99]. Other in vitro studies also proposed a crucial role of WNT5A in promoting OS cell invasiveness and migration [124,125,126,127]. An earlier study showed that Wnt inhibitory factor 1 (WIF1) is epigenetically silenced in human osteosarcoma and that targeted disruption in mice accelerates osteosarcomagenesis [120]. These studies suggest that the Wnt pathway may be an important target to treat OS. Moreover, Jag2 and Notch4 are highly expressed in some murine osteosarcomas [109], and NOTCH3 has somatic copy-number alterations in about 10% of human osteosarcoma samples [7]. However, the roles of these Notch genes in osteosarcoma are still unclear, although they have been studied in other cancers [128,129]. Interestingly, we found that 4SC-202 may contribute to NOTCH3 protein degradation but has little effect on its mRNA transcription (Figure 8B,C). Indeed, a study showed that NOTCH3 acetylation instructs its ubiquitination and proteasome-mediated degradation [130], and our previous study showed that Notch proteins can form a nuclear complex with HDAC1 in OS cells [131]. Together, our data suggest that inhibition of Wnt and Notch signaling may contribute to 4SC-202′s effect on cell invasion and migration in vitro, although our model did not provide direct in vivo evidence (Figure 9D).

Emerging evidence implies that the epigenetic state is associated with drug resistance, maintenance of cancer stem cells (CSC), and metastasis [132]. This may apply to resistance mechanisms of monotherapy using HDACi or in combination with standard-of-care therapy (MAP, consisting of methotrexate, adriamycin (doxorubicin), and platinol (cisplatin)) for OS patients. Several studies have proposed a mechanism of drug resistance using HDACi agents for osteosarcoma in which the multidrug resistance protein 1 (MDR1) encoded by the ABCB1 gene is upregulated in doxorubicin-resistant OS cells, which are also resistant to HDACi agents [80,82]. Consistent with the aforementioned studies, we report upregulation of ABCB1 by 4SC-202, implying that 4SC-202-treated OS cells may survive through this mechanism (Figure 8F). Furthermore, similarly to the MAP therapy, certain HDACi are powerful agents that kill highly proliferating OS cells but fail to suppress CSCs. In contrast, some HDACi may expand SOX2-positive CSC population [65]. Interestingly, we found that 4SC-202 downregulates expression of SOX2 (Figure 8F). So far, the mechanism by which HDACi affect the CSC population has not been fully studied. However, the inhibitory effect of 4SC-202 on SOX2-positive OS cells warrants future research on how type I HDACs regulate the maintenance of OS CSC by regulating SOX2 function. Additionally, we also found that 4SC-202 upregulates expression of MICA and ULBP1, which encode ligands for an activating receptor—NKG2D, expressed on natural killer (NK) cells—and stimulate the NK cell-mediated cytotoxicity against cancer cells, indicating that 4SC-202 may provide favorable immune-modulatory effects on cancer treatment (Figure 8G) [77,133]. In summary, our in vitro and in vivo results are promising, but further research on the potential effects of 4SC-202 alone and in combination with other drugs in the context of patient-derived xenografts (PDXs), which are assumed to closely resemble the original human tumor samples, is necessary and important [60,134,135,136]. The potential partners of 4SC-202 in combination therapy should favor agents that target autophagenesis, which promotes cancer cell survival, CSC signaling pathways such as Wnt and Notch, drug-resistant genes such as ABCB1, key components of the proteasome, and immune cells for immunotherapy.

## 5. Conclusions

We have demonstrated that 4SC-202 inhibits osteosarcoma cell growth in vitro and in vivo, and advanced our understanding of the key roles of HDAC1, HDAC2, and HDAC3 in the biological behaviors of osteosarcoma. The anti-tumor effects were featured by combined induction of cell-cycle arrest at the G2/M phase, the apoptotic program, and a reduction in the invasive and migratory ability of osteosarcoma. 4SC-202 has little capacity to promote osteogenic differentiation. 4SC-202 revised the global transcriptome and induced distinct signatures of gene expression in osteosarcoma cells in vitro. In vivo, 4SC-202 decreased tumor growth in established human tumor xenografts in immunodeficient mice. Mechanistically, we revealed key targets regulated by 4SC-202 that contribute to cell cycle, apoptosis, CSC stemness, drug resistance, immunotherapy, and canonical signaling pathways associated with the progression and metastasis of osteosarcoma. Furthermore, our data provide a rationale for further preclinical studies to access the efficacy of 4SC-202 as a second-line therapy to improve treatment options for metastatic osteosarcoma. 

## Data Availability

The raw sequence data of RNA-seq generated in this study have been stored in the National Center for Biotechnology Information (NCBI) sequence reading archive database (accession numbers: SRR14772115 to SRR14772126). The data presented in this study are available on request from the corresponding author.

## Supplementary Materials

The following are available online: https://www.mdpi.com/article/10.3390/cancers13164199/s1. Table S1: Significantly differentially expressed genes from 4SC-202-treated vs. control SJSA-1 cells. Table S2: Significantly differentially expressed genes from 4SC-202-treated vs. control hFOB 1.19 cells. Table S3: Pathway analysis of significantly differentially expressed genes from 4SC-202-treated vs. control SJSA-1 cells. Table S4: Pathway analysis of significantly differentially expressed genes from 4SC-202-treated vs. control hFOB 1.19 cells. Figure S1: Effect of 4SC-202 on cell proliferation and viability in vitro. Figure S2: Effect of 4SC-202 on acetylation of H3k27Ac protein in vitro. Figure S3: Effect of 4SC-202 on cell-cycle distribution and apoptosis in human hFOB 1.19 cells. Figure S4: Assessment of RNA quality for 12 samples applied in RNA-seq analysis. Figure S5: RNA-seq analysis of SJSA-1 and hFOB 1.19 cell lines. Figure S6: Differentially expressed genes and pathways in hFOB 1.19 cell lines.
