# Supplementary material for: Selective Targeting of Class I Histone Deacetylases in a Model of Human Osteosarcoma
Source: Cancers (Basel). 2021 Aug 20;13(16):4199. doi: 10.3390/cancers13164199 (PMC8394112; doi:10.3390/cancers13164199)
Supplement: Supplementary file 1 [file cancers-13-04199-s001.zip › cancers-1287258-original images.pdf]

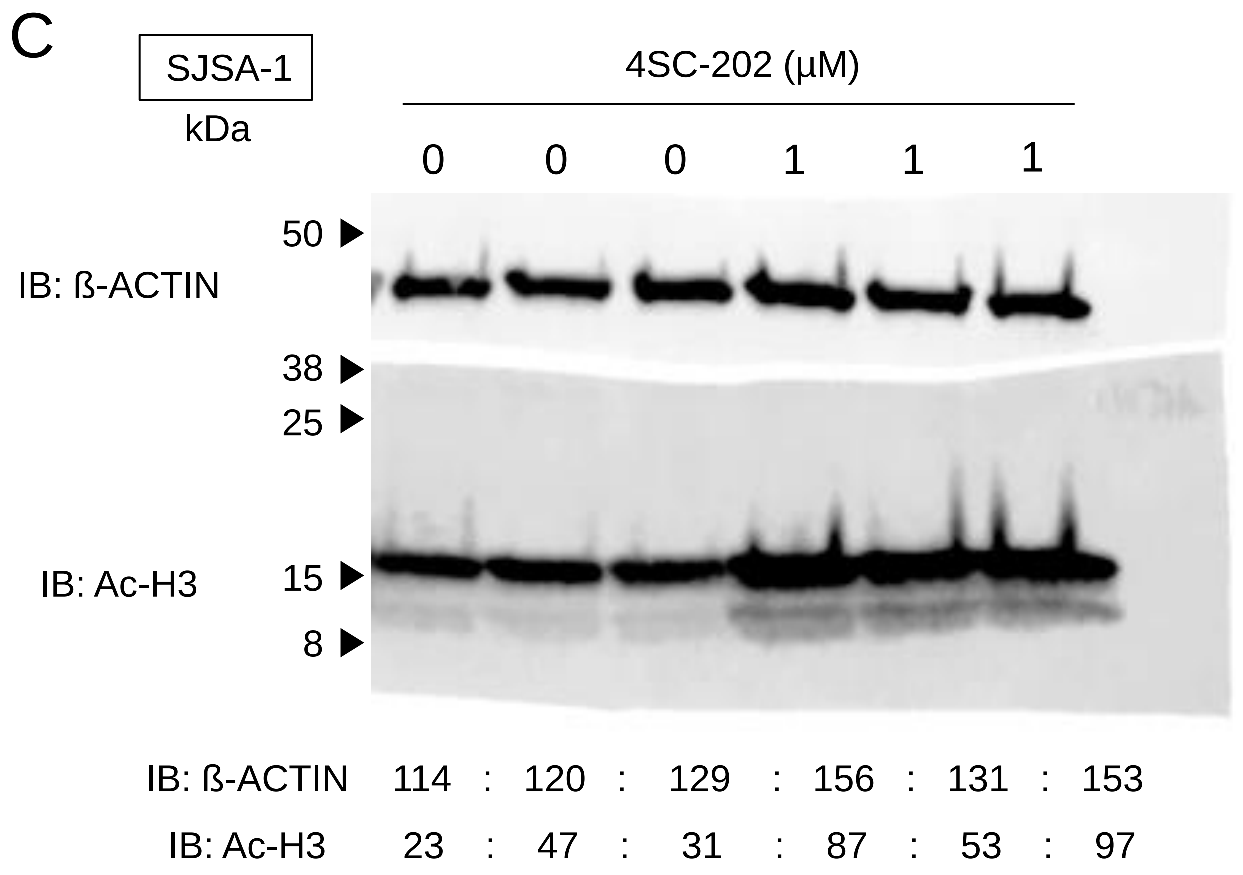
C
SJSA-1
4SC-202 (µM)
kDa
0 0 0 1 1 1
50
38
25
15
8
IB: ß-ACTIN
IB: Ac-H3
IB: ß-ACTIN 114 : 120 : 129 : 156 : 131 : 153
IB: Ac-H3 23 : 47 : 31 : 87 : 53 : 97

Fig. 1C

C

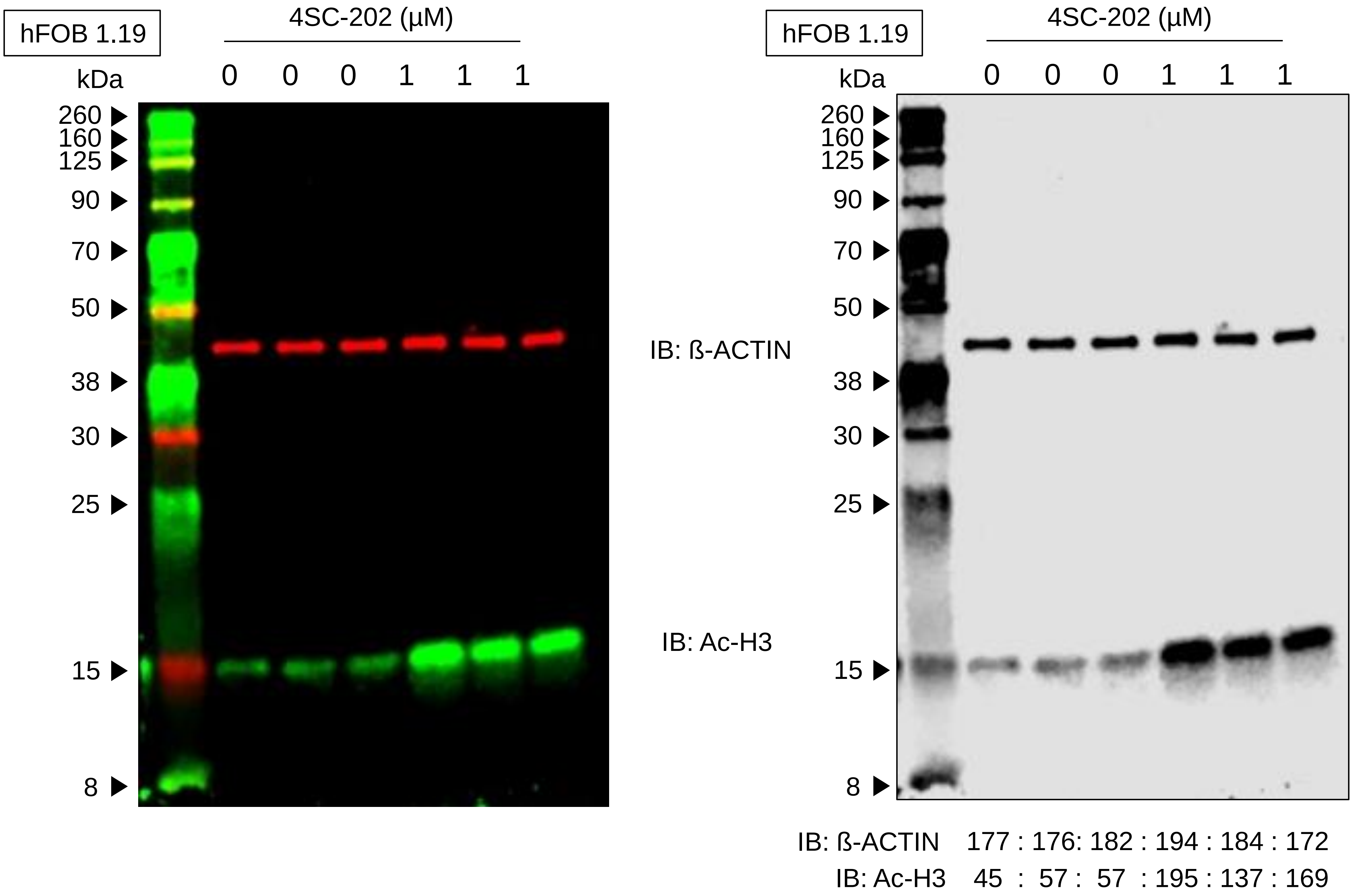
hFOB 1.19
4SC-202 (µM)
kDa
0 0 0 1 1 1
260
160
125
90
70
50
38
30
25
15
8
IB: ß-ACTIN
IB: Ac-H3
hFOB 1.19
4SC-202 (µM)
kDa
0 0 0 1 1 1
260
160
125
90
70
50
38
30
25
15
8
IB: ß-ACTIN 177 : 176: 182 : 194 : 184 : 172
IB: Ac-H3 45 : 57 : 57 : 195 : 137 : 169

Fig. 2C

C

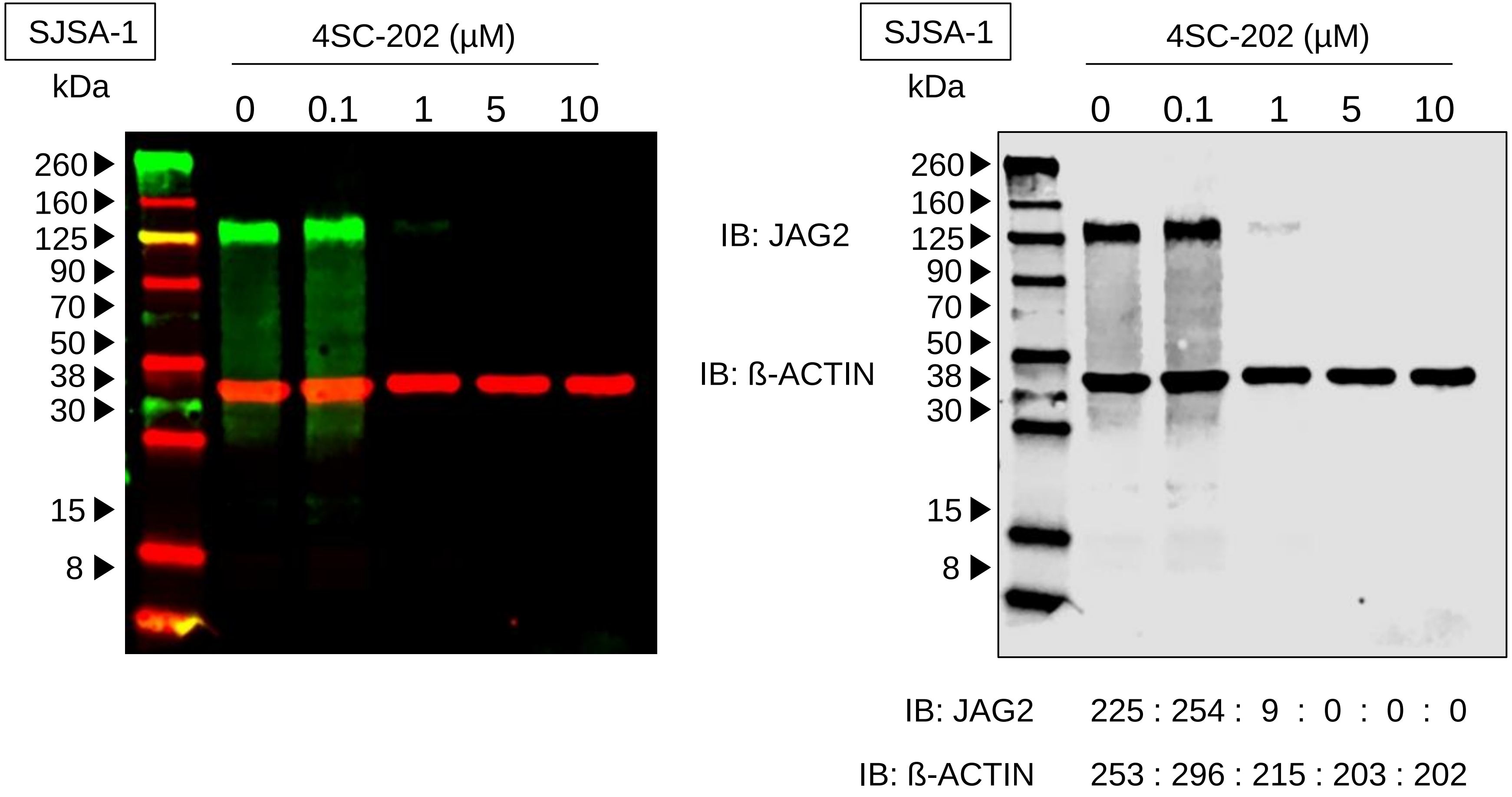
SJSA-1
4SC-202 (µM)
kDa
0
0.1
1
5
10
260
160
125
90
70
50
38
30
15
8
IB: JAG2
IB: ß-ACTIN
SJSA-1
4SC-202 (µM)
kDa
0
0.1
1
5
10
260
160
125
90
70
50
38
30
15
8
IB: JAG2 225 : 254 : 9 : 0 : 0 : 0
IB: ß-ACTIN 253 : 296 : 215 : 203 : 202

Fig. 8C

C

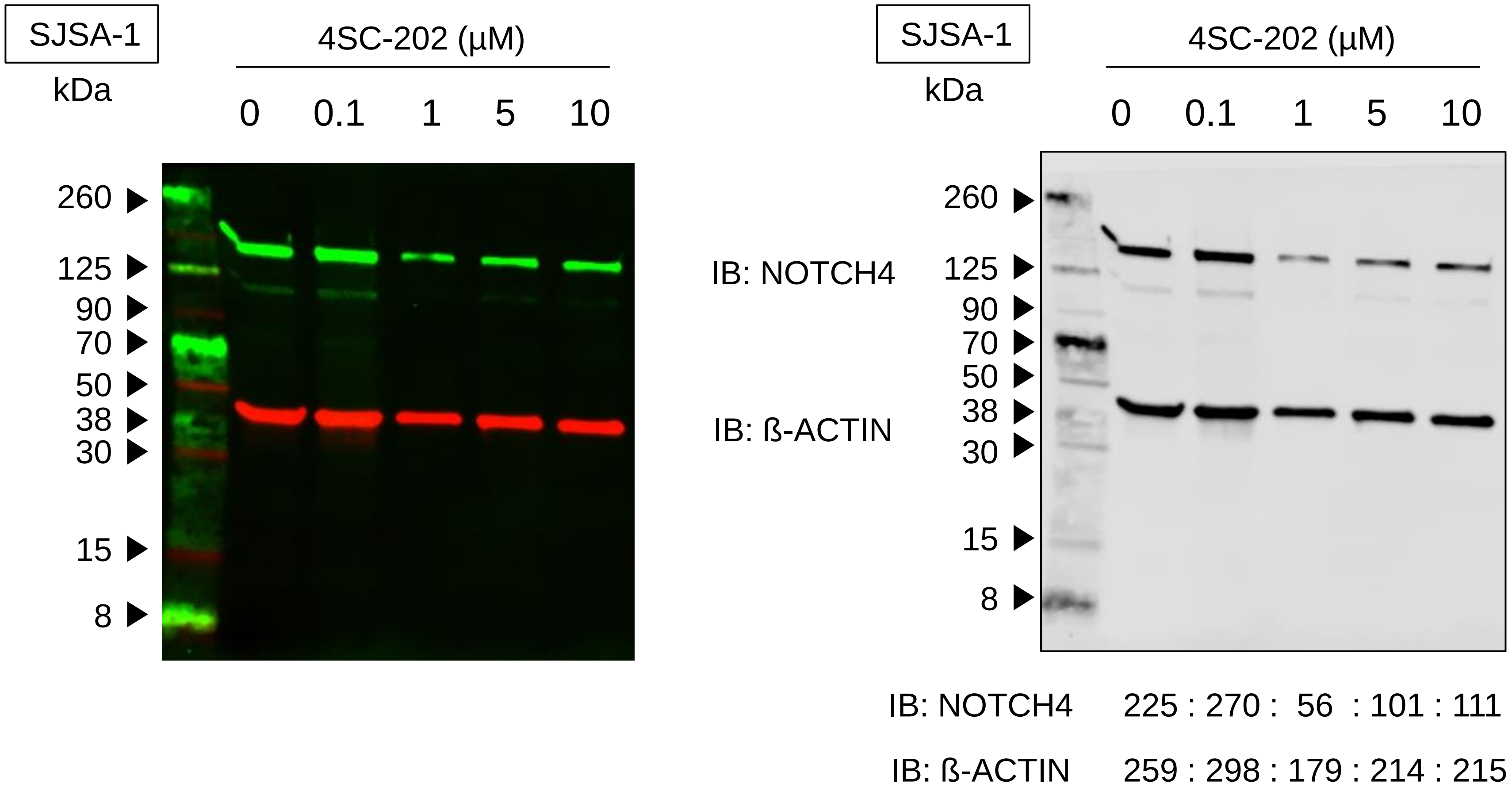
SJSA-1
kDa
4SC-202 (µM)
0
0.1
1
5
10
260
125
90
70
50
38
30
15
8
IB: NOTCH4
IB: ß-ACTIN
SJSA-1
kDa
4SC-202 (µM)
0
0.1
1
5
10
260
125
90
70
50
38
30
15
8
IB: NOTCH4 225 : 270 : 56 : 101 : 111
IB: ß-ACTIN 259 : 298 : 179 : 214 : 215

Fig. 8C

C

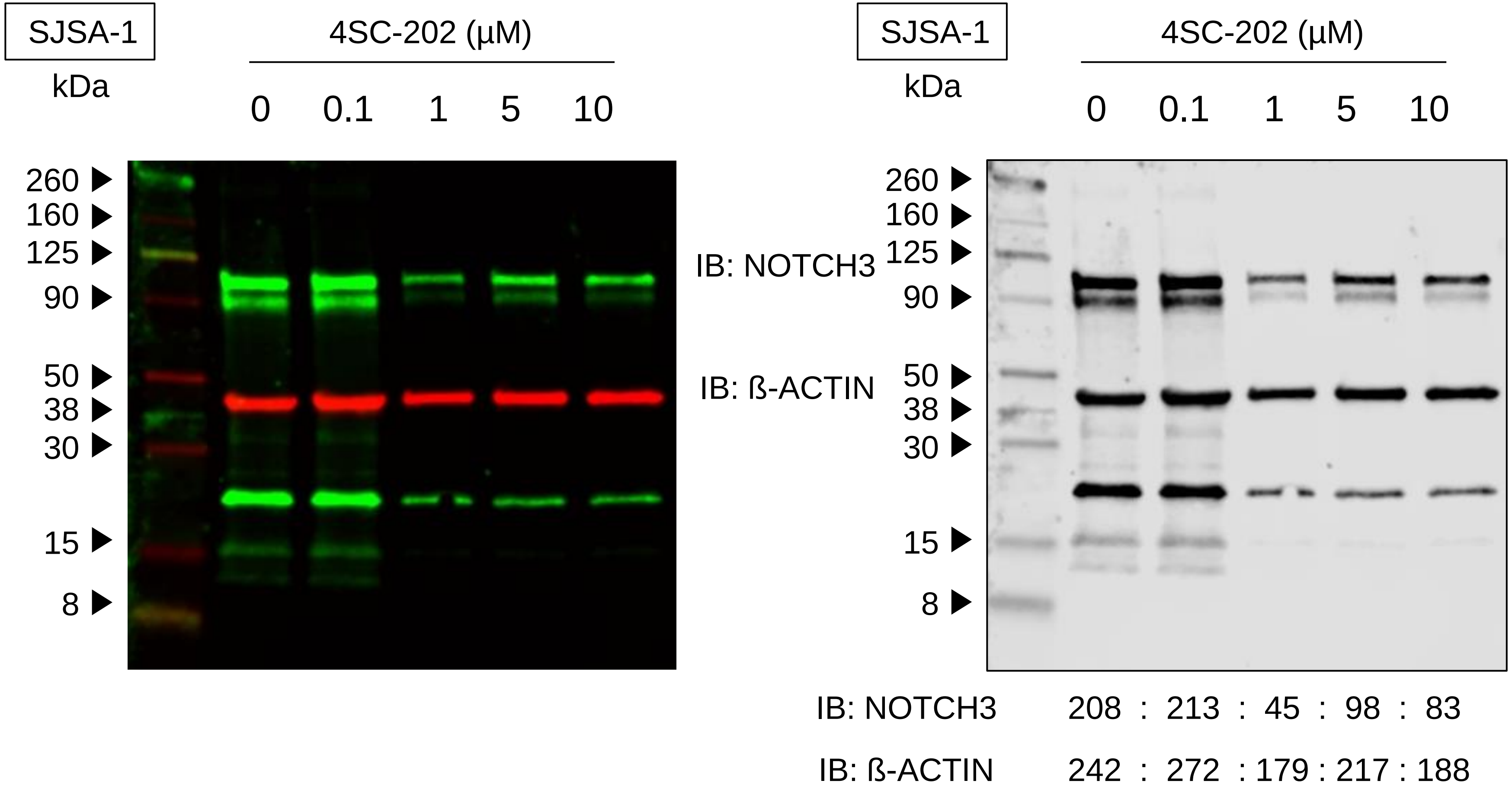

SJSA-1
kDa
4SC-202 (µM)
0
0.1
1
5
10
260
160
125
90
50
38
30
15
8
IB: NOTCH3
IB: ß-ACTIN
SJSA-1
kDa
4SC-202 (µM)
0
0.1
1
5
10
260
160
125
90
50
38
30
15
8
IB: NOTCH3 208 : 213 : 45 : 98 : 83
IB: ß-ACTIN 242 : 272 : 179 : 217 : 188

Fig. 8C

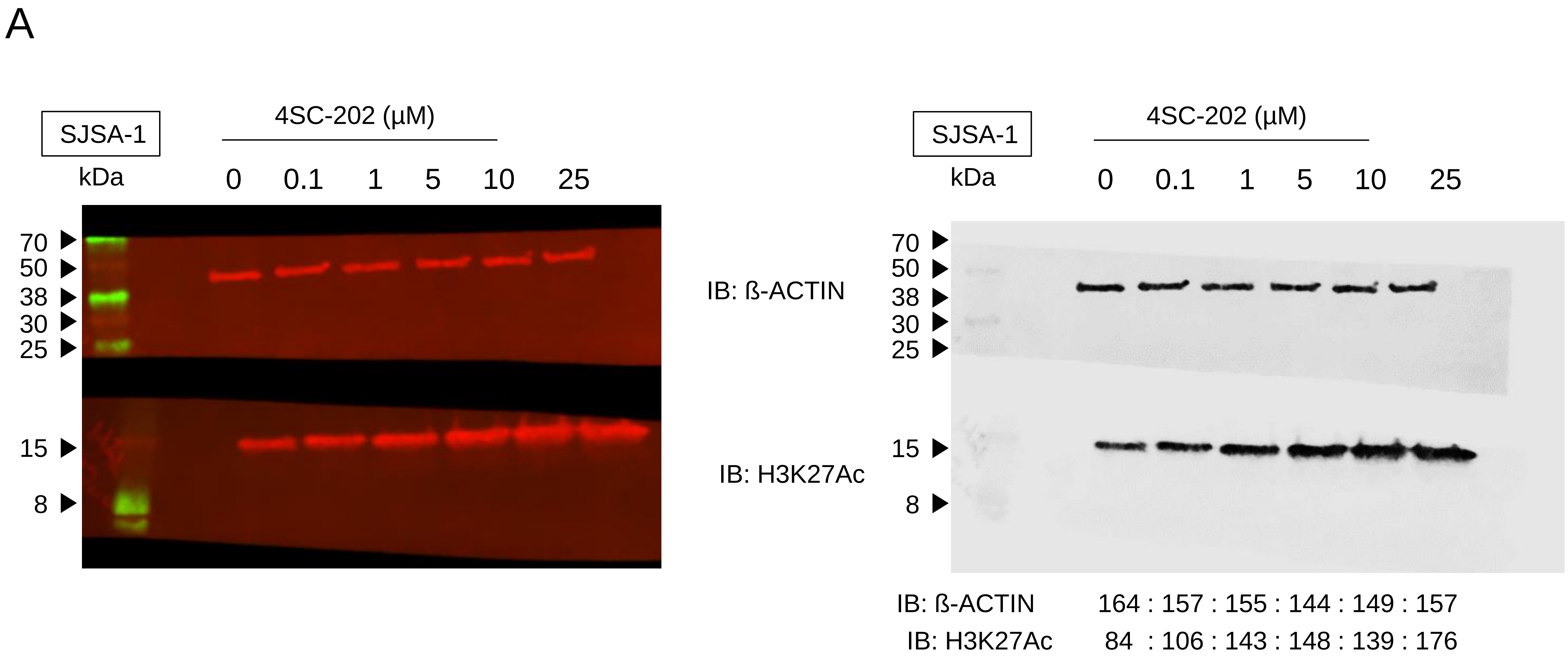
A
SJSA-1
kDa
4SC-202 (µM)
0 0.1 1 5 10 25
70
50
38
30
25
15
8
IB: ß-ACTIN
IB: H3K27Ac
SJSA-1
kDa
4SC-202 (µM)
0 0.1 1 5 10 25
70
50
38
30
25
15
8
IB: ß-ACTIN 164 : 157 : 155 : 144 : 149 : 157
IB: H3K27Ac 84 : 106 : 143 : 148 : 139 : 176

Fig. S2A

B

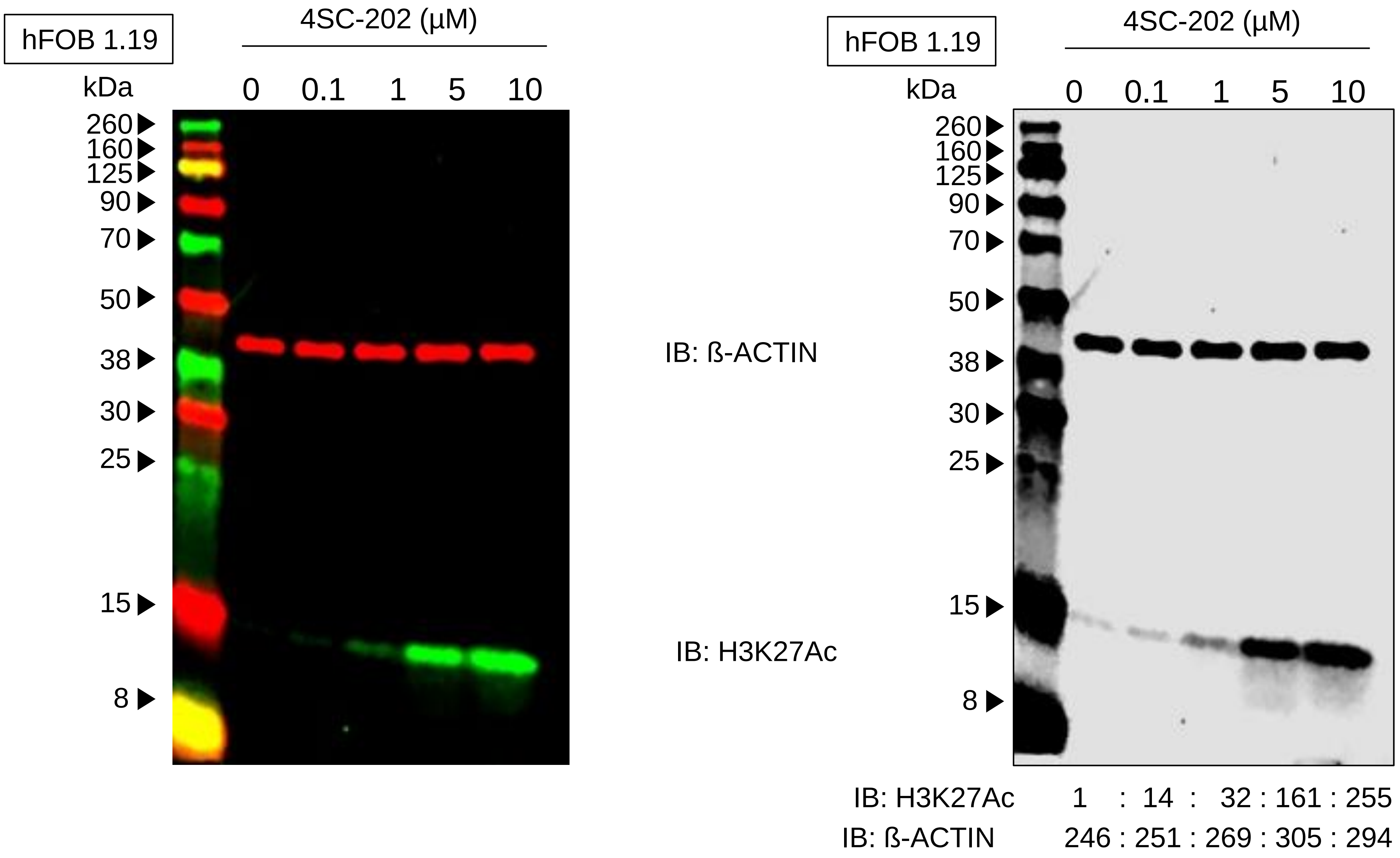
hFOB 1.19
4SC-202 (µM)
kDa
0 0.1 1 5 10
260
160
125
90
70
50
38
30
25
15
8
IB: ß-ACTIN
IB: H3K27Ac
hFOB 1.19
4SC-202 (µM)
kDa
0 0.1 1 5 10
260
160
125
90
70
50
38
30
25
15
8
IB: H3K27Ac 1 : 14 : 32 : 161 : 255
IB: ß-ACTIN 246 : 251 : 269 : 305 : 294

Fig. S2B

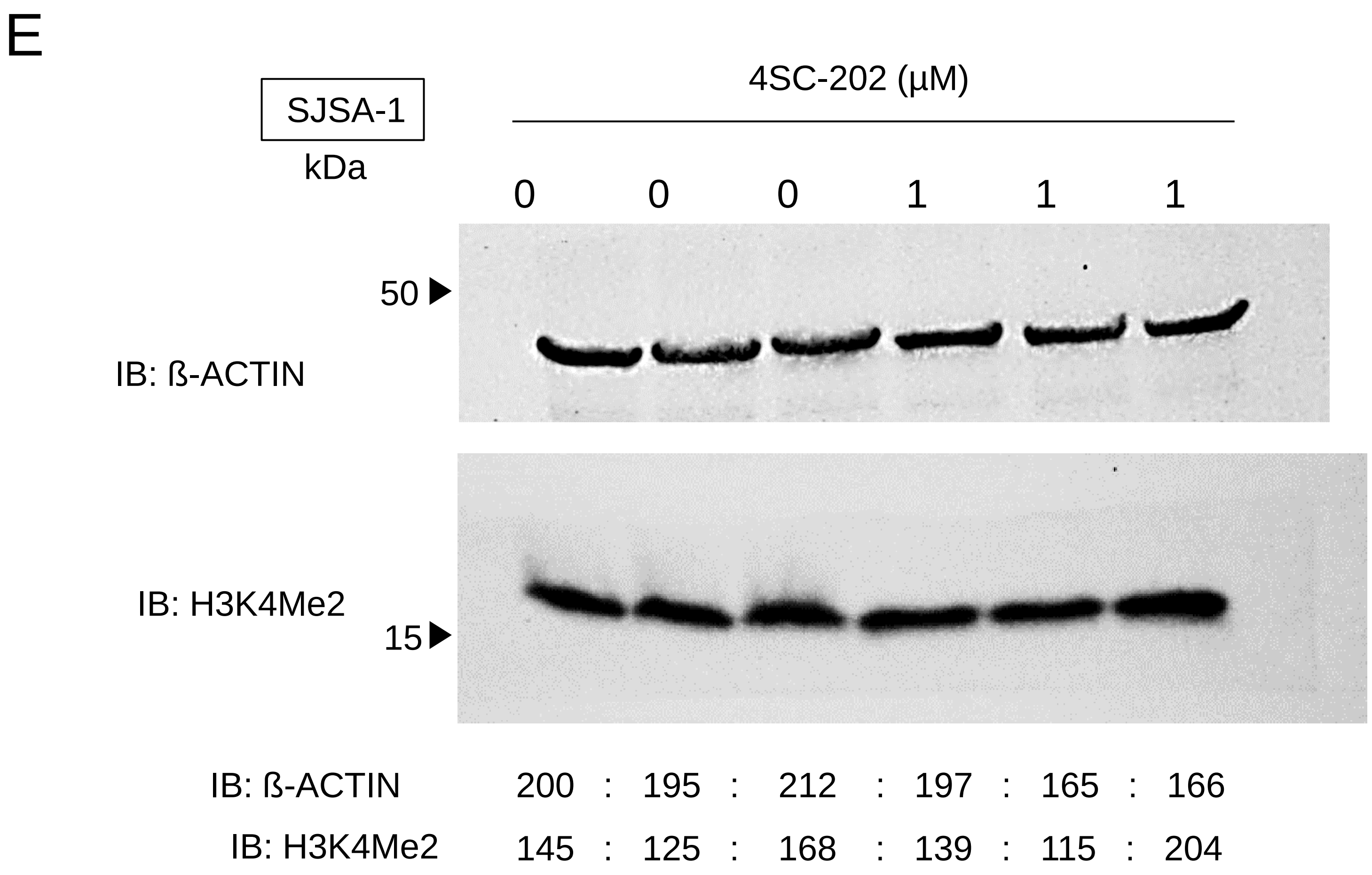
E
SJSA-1
kDa
4SC-202 (μM)
0 0 0 1 1 1
50
IB: ß-ACTIN
IB: H3K4Me2
15
IB: ß-ACTIN 200 : 195 : 212 : 197 : 165 : 166
IB: H3K4Me2 145 : 125 : 168 : 139 : 115 : 204

Fig. S2E

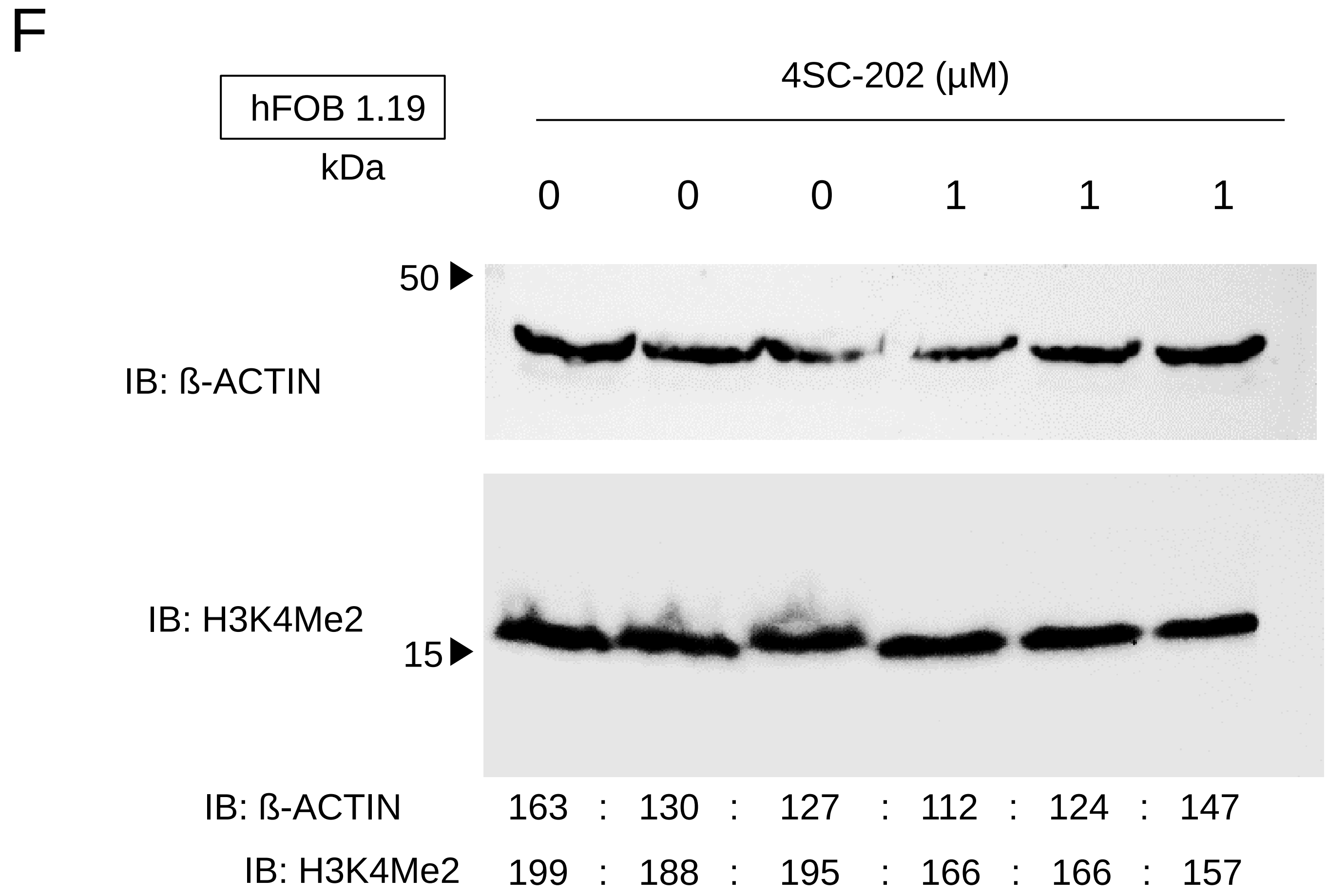
F
hFOB 1.19
kDa
4SC-202 (µM)
0 0 0 1 1 1
50
IB: ß-ACTIN
IB: H3K4Me2
15
IB: ß-ACTIN 163 : 130 : 127 : 112 : 124 : 147
IB: H3K4Me2 199 : 188 : 195 : 166 : 166 : 157

Fig. S2F
