# Supplementary material for: Selective Targeting of Class I Histone Deacetylases in a Model of Human Osteosarcoma
Source: Cancers (Basel). 2021 Aug 20;13(16):4199. doi: 10.3390/cancers13164199 (PMC8394112; doi:10.3390/cancers13164199)
Supplement: Supplementary file 1 [file cancers-13-04199-s001.zip › Supplemental Figs Torres et al.pdf]

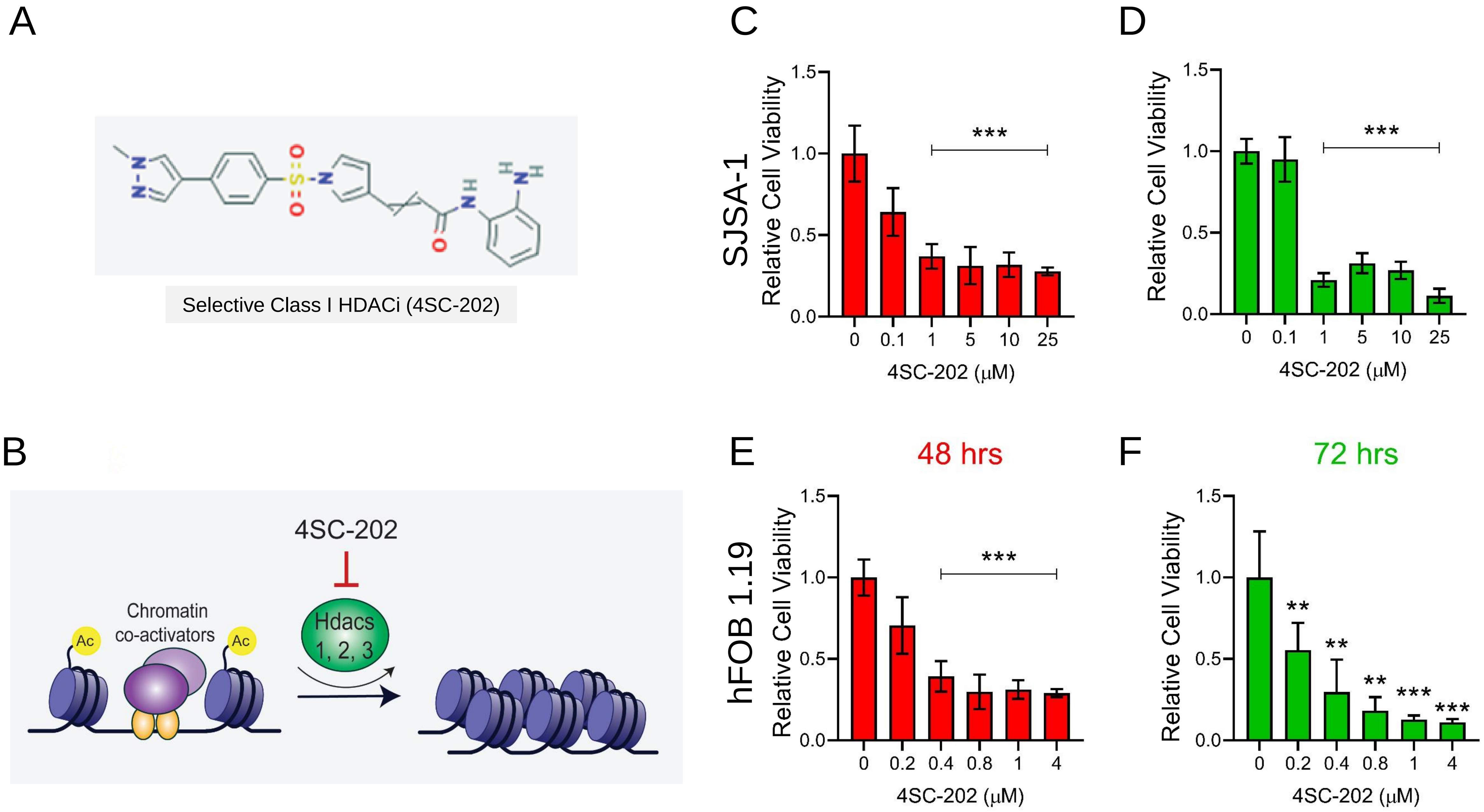
A
Selective Class I HDACi (4SC-202)
B
4SC-202
Chromatin
co-activators
Ac
Ac
Hdacs
1, 2, 3
C
SJSA-1
Relative Cell Viability
1.5
1.0
0.5
0.0
***
0
0.1
1
5
10
25
4SC-202 (μM)
D
Relative Cell Viability
1.5
1.0
0.5
0.0
***
0
0.1
1
5
10
25
4SC-202 (μM)
E
48 hrs
hFOB 1.19
Relative Cell Viability
1.5
1.0
0.5
0.0
***
0
0.2
0.4
0.8
1
4
4SC-202 (μM)
F
72 hrs
Relative Cell Viability
1.5
1.0
0.5
0.0
**
**
**
***
***
0
0.2
0.4
0.8
1
4
4SC-202 (μM)

Fig. S1

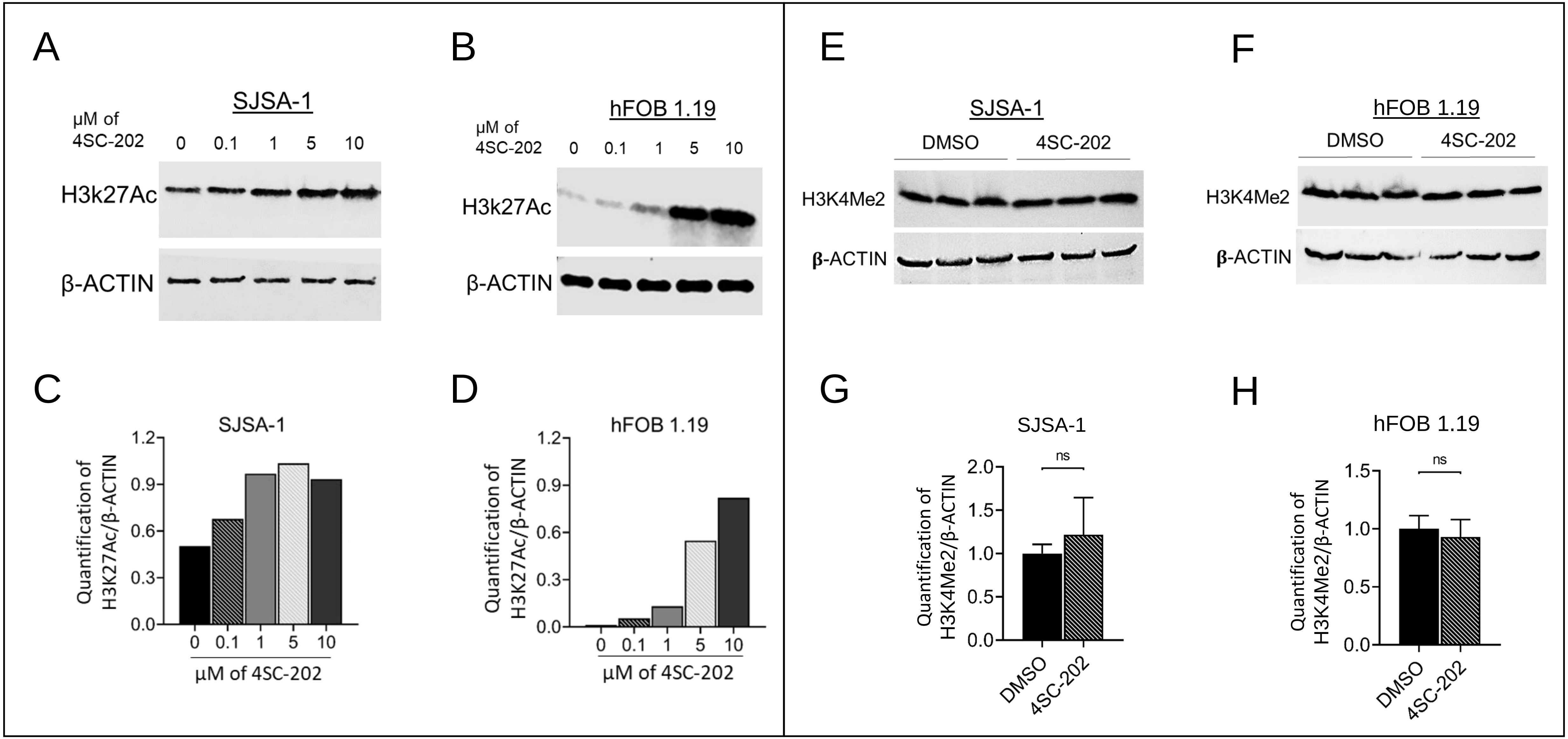
A
SJSA-1
μM of 4SC-202
0 0.1 1 5 10
H3k27Ac
β-ACTIN
B
hFOB 1.19
μM of 4SC-202
0 0.1 1 5 10
H3k27Ac
β-ACTIN
E
SJSA-1
DMSO
4SC-202
H3K4Me2
β-ACTIN
F
hFOB 1.19
DMSO
4SC-202
H3K4Me2
β-ACTIN
C
SJSA-1
Quantification of H3K27Ac/β-ACTIN
1.2
0.9
0.6
0.3
0.0
0 0.1 1 5 10
μM of 4SC-202
D
hFOB 1.19
Quantification of H3K27Ac/β-ACTIN
1.2
0.9
0.6
0.3
0.0
0 0.1 1 5 10
μM of 4SC-202
G
SJSA-1
ns
Quantification of H3K4Me2/β-ACTIN
2.0
1.5
1.0
0.5
0.0
DMSO
4SC-202
H
hFOB 1.19
ns
Quantification of H3K4Me2/β-ACTIN
1.5
1.0
0.5
0.0
DMSO
4SC-202

Fig. S2

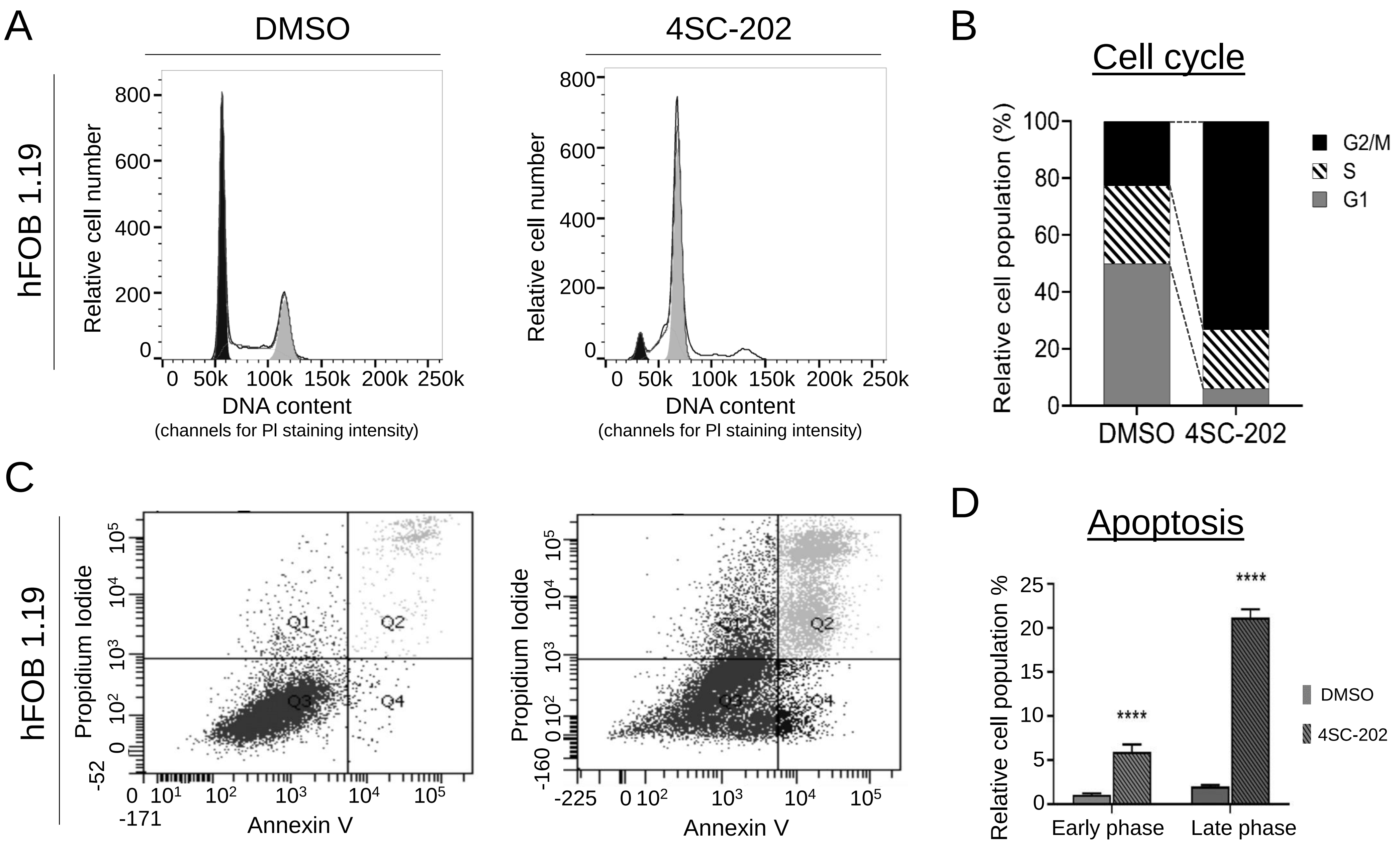

A
DMSO
4SC-202
hFOB 1.19
Relative cell number
DNA content
(channels for PI staining intensity)
B
Cell cycle
Relative cell population (%)
G2/M
S
G1
DMSO
4SC-202
C
Propidium Iodide
Annexin V
Q1
Q2
Q3
Q4
D
Apoptosis
Relative cell population %
****
DMSO
4SC-202
Early phase
Late phase

Fig. S3

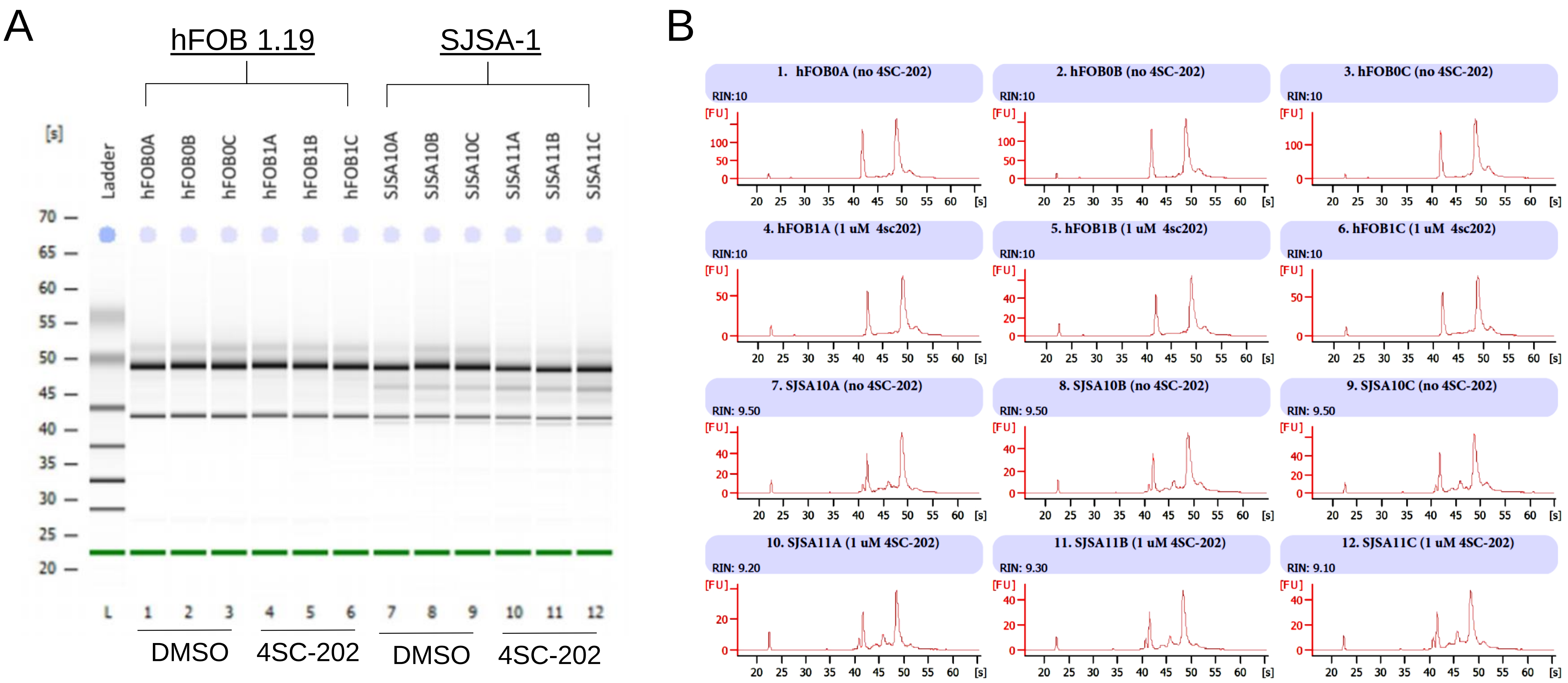
A
hFOB 1.19
SJSA-1
DMSO
4SC-202
DMSO
4SC-202
B
1. hFOB0A (no 4SC-202)
RIN:10
2. hFOB0B (no 4SC-202)
RIN:10
3. hFOB0C (no 4SC-202)
RIN:10
4. hFOB1A (1 uM 4sc202)
RIN:10
5. hFOB1B (1 uM 4sc202)
RIN:10
6. hFOB1C (1 uM 4sc202)
RIN:10
7. SJSA10A (no 4SC-202)
RIN: 9.50
8. SJSA10B (no 4SC-202)
RIN: 9.50
9. SJSA10C (no 4SC-202)
RIN: 9.50
10. SJSA11A (1 uM 4SC-202)
RIN: 9.20
11. SJSA11B (1 uM 4SC-202)
RIN: 9.30
12. SJSA11C (1 uM 4SC-202)
RIN: 9.10

Fig. S4

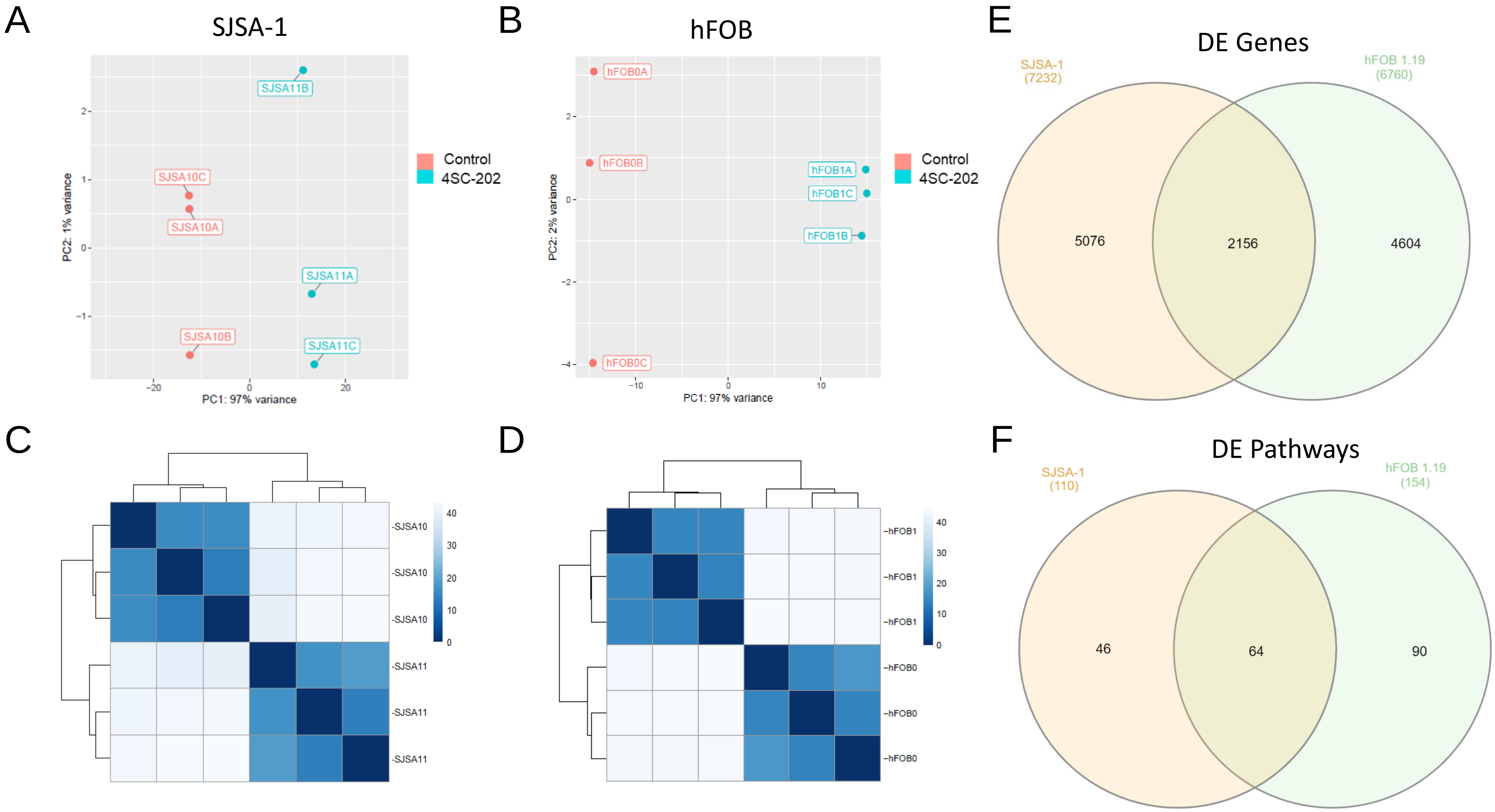
A
SJSA-1
SJSA11B
SJSA10C
SJSA10A
SJSA11A
SJSA10B
SJSA11C
PC2: 1% variance
PC1: 97% variance
Control
4SC-202
B
hFOB
hFOB0A
hFOB0B
hFOB1A
hFOB1C
hFOB1B
hFOB0C
PC2: 2% variance
PC1: 97% variance
Control
4SC-202
E
DE Genes
SJSA-1
(7232)
hFOB 1.19
(6760)
5076
2156
4604
C
SJSA10
SJSA10
SJSA10
SJSA11
SJSA11
SJSA11
D
hFOB1
hFOB1
hFOB1
hFOB0
hFOB0
hFOB0
F
DE Pathways
SJSA-1
(110)
hFOB 1.19
(154)
46
64
90
Fig. S5

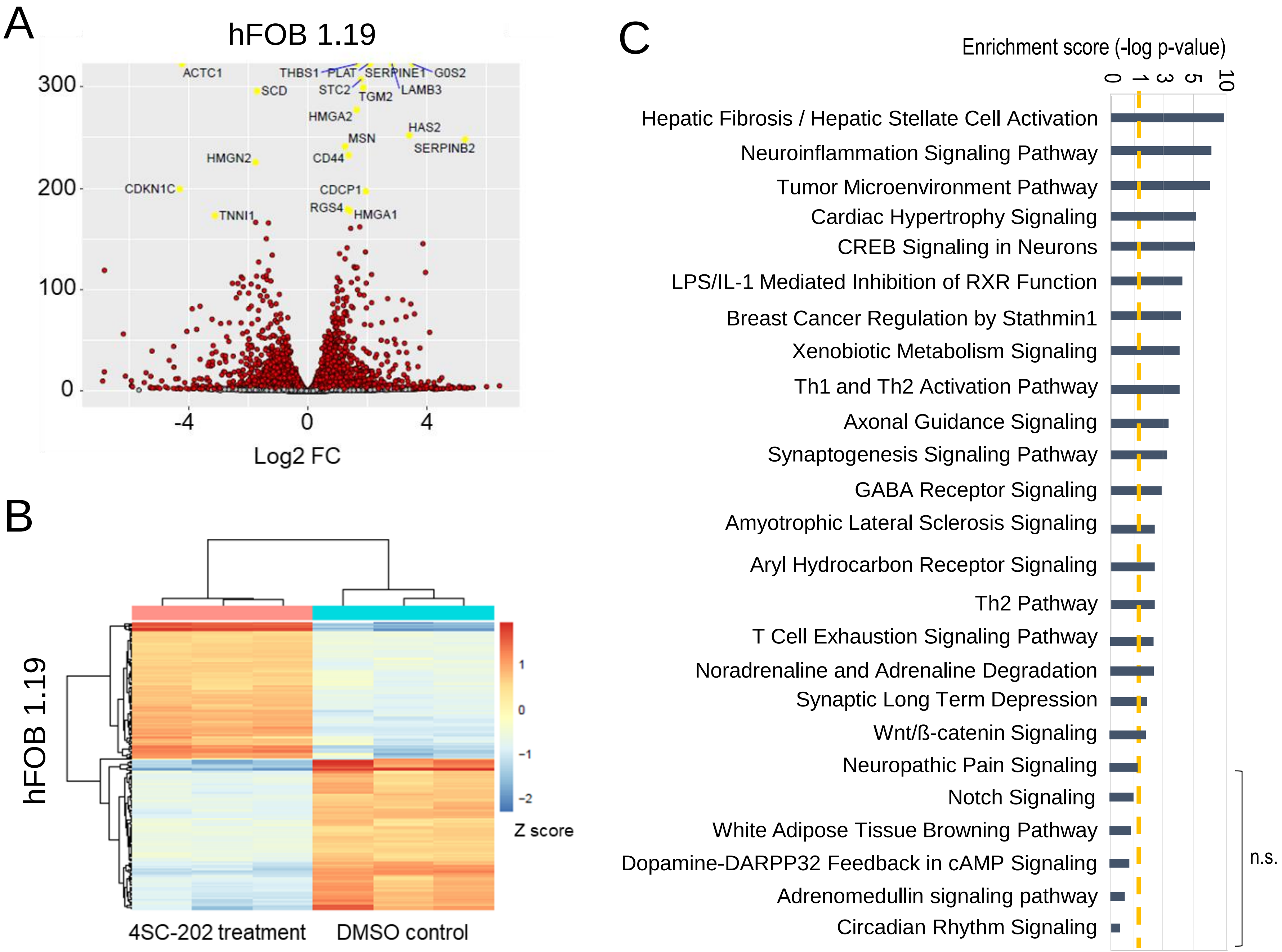
A
hFOB 1.19
ACTC1
THBS1
PLAT
SERPINE1
G0S2
SCD
STC2
TGM2
LAMB3
HMGA2
HAS2
MSN
SERPINB2
HMGN2
CD44
CDKN1C
CDCP1
RGS4
HMGA1
TNNI1
300
200
100
0
-4
0
4
Log2 FC
B
hFOB 1.19
1
0
-1
-2
Z score
4SC-202 treatment
DMSO control
C
Enrichment score (-log p-value)
0
1
3
5
10
Hepatic Fibrosis / Hepatic Stellate Cell Activation
Neuroinflammation Signaling Pathway
Tumor Microenvironment Pathway
Cardiac Hypertrophy Signaling
CREB Signaling in Neurons
LPS/IL-1 Mediated Inhibition of RXR Function
Breast Cancer Regulation by Stathmin1
Xenobiotic Metabolism Signaling
Th1 and Th2 Activation Pathway
Axonal Guidance Signaling
Synaptogenesis Signaling Pathway
GABA Receptor Signaling
Amyotrophic Lateral Sclerosis Signaling
Aryl Hydrocarbon Receptor Signaling
Th2 Pathway
T Cell Exhaustion Signaling Pathway
Noradrenaline and Adrenaline Degradation
Synaptic Long Term Depression
Wnt/ß-catenin Signaling
Neuropathic Pain Signaling
Notch Signaling
White Adipose Tissue Browning Pathway
Dopamine-DARPP32 Feedback in cAMP Signaling
Adrenomedullin signaling pathway
Circadian Rhythm Signaling
n.s.

Fig. S6
