# Supplementary material for: Selective Targeting of Class I Histone Deacetylases in a Model of Human Osteosarcoma
Source: Cancers (Basel). 2021 Aug 20;13(16):4199. doi: 10.3390/cancers13164199 (PMC8394112; doi:10.3390/cancers13164199)
Supplement: Supplementary file 1 [file cancers-13-04199-s001.zip › Supplementary figure legends.pdf]

Supplementary figure legends:

Fig. S1 Effect of 4SC-202 on cell proliferation and viability *in vitro*.

(A), Chemical structure of 4SC-202 (modified from https://www.selleckchem.com/). (B), Simplified schematic of the epigenetic mechanism of action for 4SC-202. Effect of 4SC-202 on cell proliferation and viability treated with increased concentration of 4SC-202 in SJSA-1 cells for (C), 24 hr; (D), 72 hr; and in hFOB 1.19 cells, (E), 24 hr; (F), 72 hr. Number of asterisks indicates level of statistical significance between groups. * $p < 0.05$, ** $p < 0.01$, *** $p < 0.001$. Data are presented as mean (SD).

Fig. S2 Effect of 4SC-202 on acetylation of H3K27Ac protein *in vitro*.

Representative western blot analysis of H3K27Ac and β-ACTIN of DMSO treated and 4SC-202 treated conditions in (A), SJSA-1 cells; (B) hFOB 1.19 cells. Densitometric quantification of the blots of protein band intensity of Ac-H3 normalized to housekeeping β-actin bands for (C), SJSA-1 cells; (D) hFOB 1.19 cells. Representative western blot analysis of H3K4Me2 and β-ACTIN of DMSO treated and 4SC-202 treated conditions in (E), SJSA-1 cells; (F) hFOB 1.19 cells. Densitometric quantification of the blots of protein band intensity of H3K4Me2 normalized to housekeeping β-actin bands for (G), SJSA-1 cells; (H) hFOB 1.19 cells.

Fig. S3. Effect of 4SC-202 on the cell cycle distribution and apoptosis in human hFOB 1.19 cells.

(A) Representative cell cycle flow cytometry profiles of cells treated with or without 4SC-202 for 24 hr. The DNA content of cells was analyzed by flow cytometry after staining with propidium iodide. (B), Relative cell population quantified data from cell-cycle profiles in A. (C), Representative flow cytometry scatter plots of cells treated with or without 4SC-202 for 24 hr and stained with annexin V (positive for apoptotic cells) and propidium iodide. (D), Quantification of apoptotic profiles for relative cell population from C. ** $p < 0.01$. Data are presented as mean (SD).

Fig. S4. Assessment of RNA quality for 12 samples applied in RNA-seq analysis.

(A), A representative gel image of RNA electrophoresis of 12 samples produced by a Bioanalyzer. L, Ladder. (B), Electropherograms of the gel image showing the RNA quality for hFOB 1.19 and SJSA-1 triplicate samples treated in absence (No. 1-3, 7-9) or presence of (No. 4-6, 10-12) of 1

μM 4sc202. All samples showed the RNA Integrity Number (RIN) higher than 9.00, confirming the RNA integrity and quality.

Fig. S5. RNA-seq analysis of SJSA-1 and hFOB 1.19 cell lines.

Principle component analysis (PCA) plot displaying distance between samples treated with or without 4SC-202 in (A), SJSA-1 cells; (B), hFOB 1.19 cells. Heatmap of Euclidian distances between samples (n = 12) treated with or without 4SC-202 in (C), SJSA-1 cells; (D), hFOB 1.19 cells. (E), Venn diagram showing the number of genes significantly regulated in treated SJSA-1 cells (n= 7195, pink color), or treated hFOB 1.19 cells (n=6740, blue color) or overlapping (n=3534) between two cell lines. (F), Venn diagram showing the number of pathways significantly altered in treated SJSA-1 cells (n= 110, pink color), or treated hFOB 1.19 cells (n=154, blue color) or overlapping (n=64) between two cell lines.

Fig. S6. Differentially expressed genes and pathways in hFOB 1.19 cell lines.

(A), A volcano plot of gene expression from RNA-seq analysis between the vehicle control and 4SC-202 treated hFOB 1.19 cells. The top 20 significantly differentially expressed genes are labeled. (B), Unsupervised hierarchical cluster analysis and heatmap representation of differentially expressed genes in hFOB 1.19 cells treated with either 1 μM 4SC-202 or DMSO for 24 hr. Intensity of color indicates expression levels (red, high; blue, low). Each column indicates a distinct sample, and each row indicates an individual gene. (C), Ingenuity pathways analysis (IPA) of cancer canonical signaling pathways associated with significantly regulated genes ($p < 0.05$) in 4SC-202-treated samples compared to the vehicle-treated samples.
